# In vivo AAV9-*Myo7a* gene rescue restores hearing and cholinergic efferent innervation in inner hair cells

**DOI:** 10.1172/jci.insight.182138

**Published:** 2024-12-06

**Authors:** Andrew P. O’Connor, Ana E. Amariutei, Alice Zanella, Sarah A. Hool, Adam J. Carlton, Fanbo Kong, Mauricio Saenz-Roldan, Jing-Yi Jeng, Marie-José Lecomte, Stuart L. Johnson, Saaid Safieddine, Walter Marcotti

**Affiliations:** 1 School of Biosciences, University of Sheffield, Sheffield, United Kingdom.; 2 Université Paris Cité, Institut Pasteur, AP-HP, INSERM, Fondation Pour l’Audition, Institut de l’Audition, IHU reConnect, F-75012 Paris, France.; 3 Neuroscience Institute, University of Sheffield, Sheffield, United Kingdom.

**Keywords:** Aging, Neuroscience, Gene therapy, Genetic diseases, Mouse models

## Abstract

In the mammalian cochlea, sensory hair cells are crucial for the transduction of acoustic stimuli into electrical signals, which are then relayed to the central auditory pathway via spiral ganglion neuron (SGN) afferent dendrites. The SGN output is directly modulated by inhibitory cholinergic axodendritic synapses from the efferent fibers originating in the superior olivary complex. When the adult cochlea is subjected to noxious stimuli or aging, the efferent system undergoes major rewiring, such that it reestablishes direct axosomatic contacts with the inner hair cells (IHCs), which occur only transiently during prehearing stages of development. The trigger, origin, and degree of efferent plasticity in the cochlea remains largely unknown. Using functional and morphological approaches, we demonstrate that efferent plasticity in the adult cochlea occurs as a direct consequence of mechanoelectrical transducer current dysfunction. We also show that, different from prehearing stages of development, the lateral olivocochlear — but not the medial olivocochlear — efferent fibers are those that form the axosomatic synapses with the IHCs. The study also demonstrates that in vivo restoration of IHC function using AAV-*Myo7a* rescue reestablishes the synaptic profile of adult IHCs and improves hearing, highlighting the potential of using gene-replacement therapy for progressive hearing loss.

## Abstract 2

<span>We report that mechanoelectrical transduction incrucial for maintaining <span>cochlear </span>IHC innervation and that the adult cochlea is amenable to AAV-based gene replacement therapy.</span>

## Introduction

Inner hair cells (IHCs), the main sensory receptors of the mammalian cochlea, are responsible for converting mechanically induced acoustic stimuli into electrical signals that are relayed via spiral ganglion neuron (SGN) afferent fibers toward the higher auditory pathway (1, 2). Mechanoelectrical transduction is performed by the stereociliary hair bundles found on the upper surface of IHCs. The displacement of the hair bundle opens mechanosensitive ion channels that are localized to the tips of the shorter 2 rows of stereocilia (3). Extracellular filaments called tip links, which span across 2 adjacent stereocilia in different rows, are required for gating the mechanoelectrical transducer (MET) channels by transmitting force from the mechanical displacement of the stereociliary bundles to the MET channel complex (4, 5). A key protein required for mechanoelectrical transduction is MYO7A, an unconventional myosin that is highly expressed in the stereocilia from very early stages of development (6, 7). MYO7A is specialized in the transport of actin-regulatory proteins and other factors to the tip of actin-based protrusions (8). Variants in the gene encoding for MYO7A cause syndromic (Usher syndrome type 1B) or nonsyndromic deafness in humans (9–11). Variants in the orthologous gene in mice, *Shaker-1* (12), also lead to profound deafness (13, 14). Interestingly, the KO of *Myo7a* in the mature cochlea has been shown to progressively abolish the MET current, and the IHCs lose most of their mature-like biophysical and morphological characteristics and reestablish inhibitory cholinergic efferent synapses (15).

In the adult cochlea, the efferent pathway that originates in the brainstem performs several important functions, including the maintenance of cochlear sensitivity in noisy environments, protecting the hair cells from noise insults, by reducing their sensitivity and modulating selective attention (16). The efferent innervation is subject to several changes during cochlear development. Before the onset of hearing, which normally occurs at around P12–P13 in mice (17, 18), IHCs exhibit axo-somatic synaptic connections with medial olivocochlear (MOC) cholinergic fibers (19). However, between the second and third postnatal weeks, IHCs lose these axo-somatic synapses, and instead, the lateral olivocochlear (LOC) fibers form axo-dendritic synapses with the SGN afferent terminals below the IHCs (20). During this time, IHCs also downregulate the postsynaptic α9α10 nAChRs (α9α10nAChRs) and SK2 channels, such that, by about P16, they are no longer able to respond to acetylcholine (21–23). The efferent system has been shown to reestablish direct axo-somatic synaptic connections with the IHCs in the aged cochlea, and also in young adult animals when MYO7A is absent (15, 24–26), suggesting that it could be triggered by some dysfunction of the hair cell MET apparatus. However, functional evidence linking the presence of the MET and the changes in the efferent system in mature IHCs is lacking. It is also not known whether the efferent reinnervation of adult IHCs of *Myo7a*-deficient mice can be undone and reverted to its normal configuration by reinstating normal IHC function, which may have implications for future therapeutic intervention aimed at treating age-related hearing loss.

In this study, we have used conditional transgenic mice to manipulate the expression of proteins involved in the IHC MET apparatus and exocytotic machinery, and we demonstrated that the rewiring of mature IHCs occurs in response to MET channel dysfunction. We also found that the postsynaptic SK2 channels and efferent synaptic currents were present in IHCs a couple of days after the reexpression of α9α10nAChRs. Finally, in vivo gene therapy with dual-AAV9-*Myo7a* partially restored hearing function in *Myo7a-*deficient mice and reestablished normal efferent innervation of adult IHCs.

## Results

### Postnatal deletion of Myo7a leads to early-onset hearing loss.

**Figure 1:**
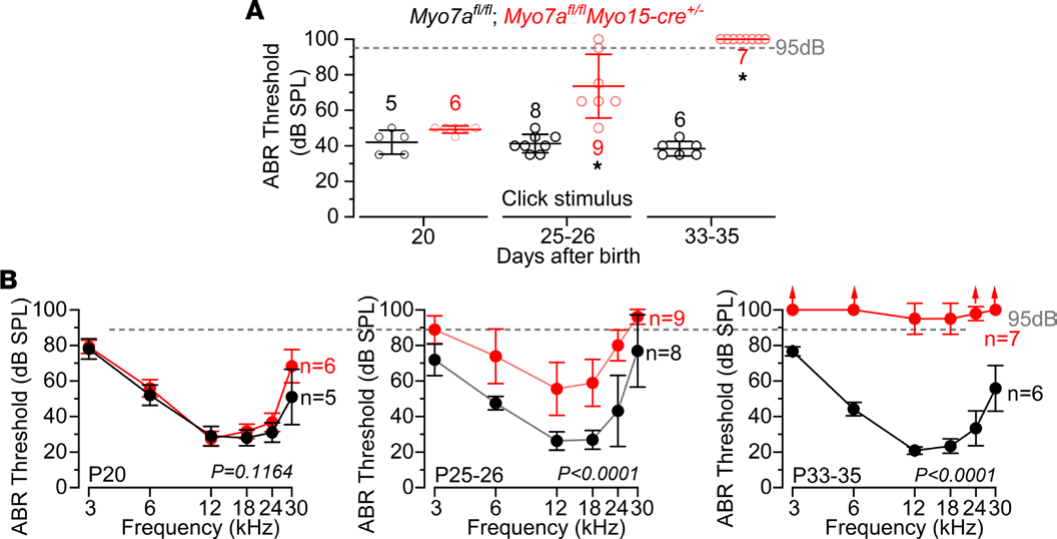
Auditory brainstem response thresholds in control and *Myo7a^fl/fl^ Myo15-cre^+/–^* mice. (**A**) Average ABR thresholds for click stimuli recorded from control *Myo7a^fl/fl^* (black) and *Myo7a^fl/fl^ Myo15-cre^+/–^* (red) male and female mice at P20, P25–P26, and P33–P35 The age group tested are shown on the *x* axis. *P < 0.0001, Tukey’s post hoc test (1-way ANOVA). (**B**) ABR thresholds for frequency-specific pure tone burst stimuli at 3, 6, 12, 18, 24, and 30 kHz recorded from controls (*Myo7a^fl/fl^*) and littermate *Myo7a^fl/fl^ Myo15-cre^+/–^* mice. Significance found using 2-way ANOVA. The number of mice tested for each genotype are shown next to the data, while the dashed lines indicate the upper threshold limit selected for these recordings (95 dB). Data are shown as mean ± SD.

Targeted postnatal deletion of *Myo7a* was achieved by crossing *Myo7a* floxed mice (*Myo7a^fl/fl^*) with *Myo15-cre^+/–^* mice (15, 27). Using auditory brainstem responses (ABRs) to assess auditory function, we found that hearing loss in *Myo7a^fl/fl^ Myo15-cre^+/–^* mice is progressive (Figure 1) and started several days following the induced downregulation of MYO7A from the IHCs (Supplemental Figure 1; supplemental material available online with this article; https://doi.org/10.1172/jci.insight.182138DS1). ABR thresholds for clicks were significantly elevated in *Myo7a^fl/fl^ Myo15-cre^+/–^* compared with control mice at P25–P26 and P33–P35 (*P* < 0.0001 for both comparisons), but not at P20 (*P* > 0.9999, Tukey’s post hoc test, 1-way ANOVA; Figure 1A). Similar findings were also observed for pure-tone evoked ABRs (Figure 1B). Compared with controls, littermate *Myo7a^fl/fl^ Myo15-cre^+/–^* mice exhibited significantly elevated ABR thresholds only at the 2 older ages (*P* < 0.0001, for both comparisons; P20: *P* = 0.1164, Tukey’s post hoc test, 2-way ANOVA; Figure 1B). The amplitude and latency of ABR wave 1 at 12 kHz, which is the frequency region that more closely matches that used for the ex vivo experiments described below, became significantly affected by the lack of MYO7A at the 2 older age ranges tested (Supplemental Figure 2). These results show that, in the absence of MYO7A, mice progressively lose their hearing (Figure 1), which is linked to the loss of the mechanoelectrical transduction (Supplemental Figure 3).

### Time course of efferent synapse formation in IHCs from Myo7a^fl/fl^ Myo15-cre^+/–^ mice.

Axosomatic efferent cholinergic synapses with IHCs are normally present only during prehearing stages (21–23, 28) or in aging mice (24–26). In this condition, the release of acetylcholine (ACh) from the efferent terminals leads to the activation of α9α10 nicotinic ACh receptors (nAChRs) on the postsynaptic IHC membrane. Since α9α10nAChRs are permeable to Ca^2+^ and are closely colocalized with the small conductance Ca^2+^ activated K^+^ channels (SK2), the release of ACh from efferent terminals causes IHC hyperpolarization (21–23). The *Myo7a^fl/fl^ Myo15-cre^+/–^* mouse strain recapitulates the efferent rewiring of the aged cochlea in young adult IHCs (15).

**Figure 2:**
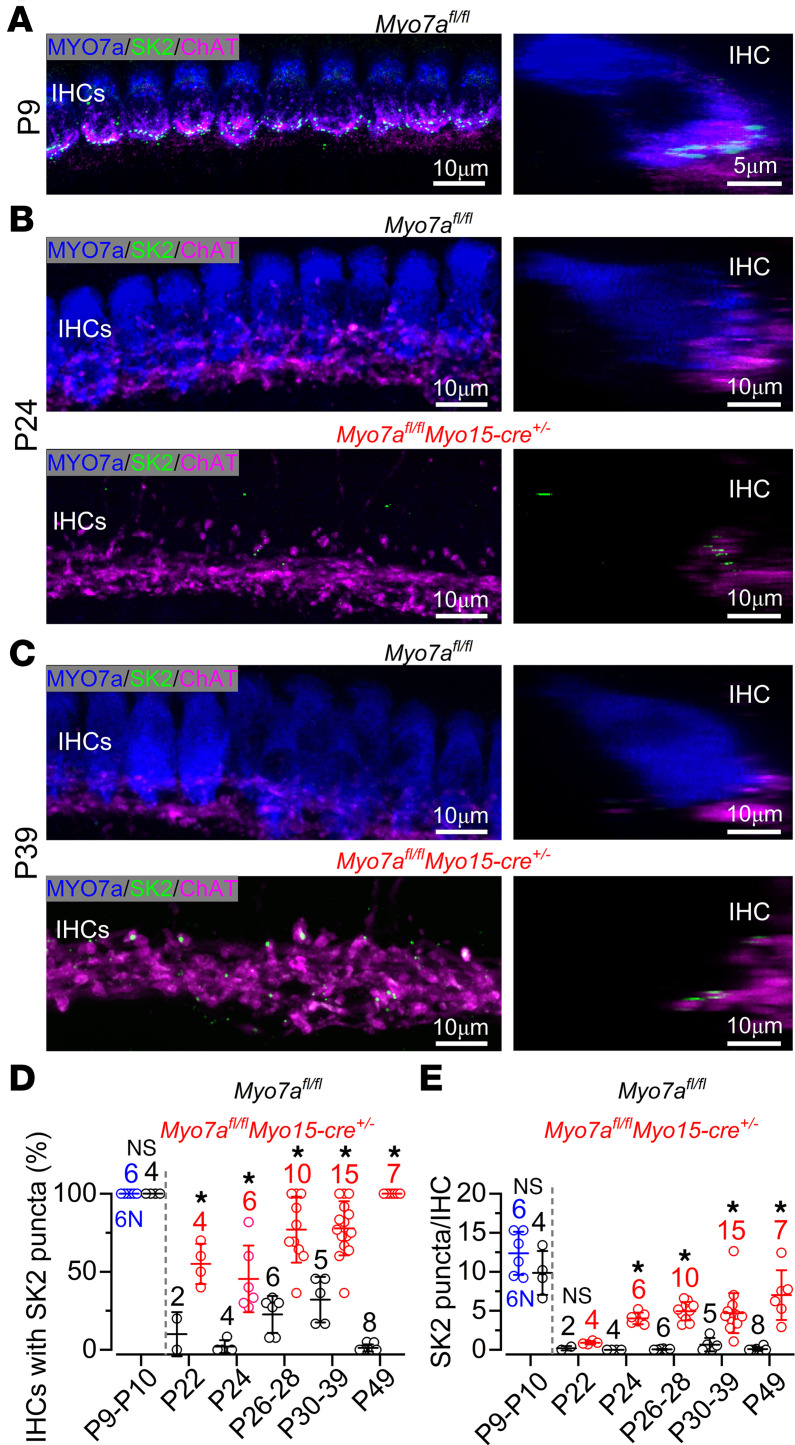
Efferent synapses return to adult IHCs in Myo7a^fl/fl^ Myo15-cre^+/–^ mice. (A–C) Maximum intensity projections of confocal *Z* stack images taken from the 9–12 kHz cochlear region in control *Myo7a^fl/fl^* (P9 [**A**], P24 [**B**], P39 [**C**]) and littermate *Myo7a^fl/f^Myo15-cre^+/–^* mice (P24 [**B**], P39 [**C**]). Cochleae were labeled with antibodies against SK2 (green), the presynaptic efferent marker ChAT (magenta), and the hair cell marker MYO7A (blue). The right panels in **A**–**C** show a single IHC rotated on the *y*, *z* plane, providing a lateral view of the IHCs, which show the juxtaposed SK2 puncta and ChAT labeling of the efferent synapses. Scale bars: 10 μm. (**D** and **E**) Percentage of IHCs that expressed SK2 puncta (**D**) and number of SK2 puncta per IHC (**E**) in 150 μm of the apical cochlea region at different age groups of both control *Myo7a^fl/fl^* and *Myo7a^fl/fl^ Myo15-cre^+/–^*. At prehearing ages, P10 WT C57BL/6N mice were also used as a comparison with P9 *Myo7a^fl/fl^*. Data are shown as mean ± SD. The number of mice used for each age group is indicated above the single data points/averages. *P < 0.05, Šídák post hoc test (2-way ANOVA).

We initially looked at changes in the expression of the postsynaptic SK2 channels in the IHCs located within 150 μm of the apical-coil sensory epithelium (9–12 kHz frequency region) using immunostaining (Figure 2, A–E). As expected, SK2 channels were expressed in all prehearing IHCs from both control *Myo7a^fl/fl^* (P9, black symbols; Figure 2, A, D, and E) and WT C57BL/6N mice (P10, blue symbols; Figure 2, D and E) but were downregulated in adult mice (Figure 2, B–D). In *Myo7a^fl/fl^ Myo15-cre^+/–^* mice, both the percentage of IHCs expressing SK2 (Figure 2D) and the number of SK2 puncta per IHC (Figure 2E) were significantly increased compared with littermate controls (*P* < 0.0001 for both comparisons, 2-way ANOVA). By P49, all the IHCs in *Myo7a^fl/fl^ Myo15-cre^+/–^* mice reexpressed SK2 channels as in prehearing cells (Figure 2D), and the number of SK2 puncta per IHC was no longer significantly different than that of control prehearing IHCs (*P* = 0.1874). Similar to immature IHCs, the reexpressed SK2 puncta were juxtaposed to efferent terminals (ChAT immunoreactivity). Although a larger number of IHCs from P22 *Myo7a^fl/fl^ Myo15-cre^+/–^* mice seem to show SK2 puncta (*P* = 0.0057, Sidak’s post hoc test, 2-way ANOVA; Figure 2D), their number per IHC was not significantly different to that from control IHCs (*P* = 0.9936; Figure 2E). This indicates that SK2 channels start to be significantly upregulated in IHCs in *Myo7a^fl/fl^ Myo15-cre^+/–^* mice older than P22 and became more pronounced over the following days (Figure 2E).

**Figure 3:**
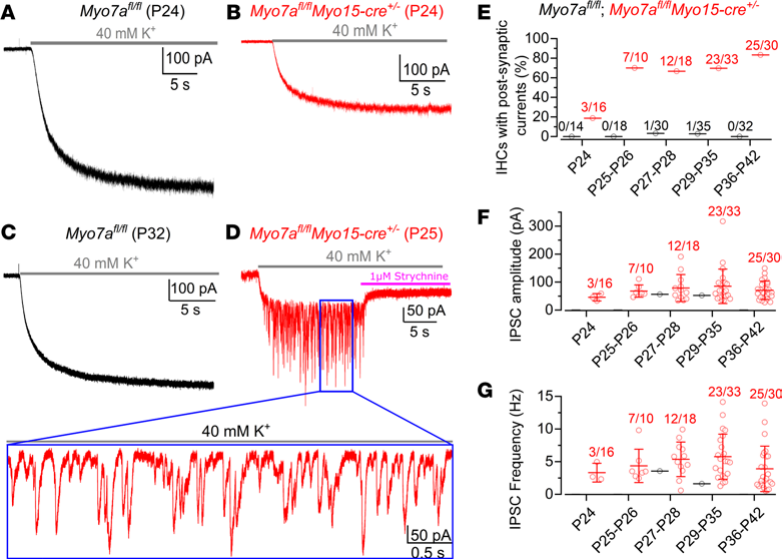
Reestablishment of efferent synapses onto the IHCs of Myo7a^fl/fl^ Myo15-cre^+/–^ mice. (**A**–**D**) Inward membrane currents recorded from IHCs of *Myo7a^fl/fl^* (P24 [**A**]; P32 [**C**]) and littermate *Myo7a^fl/fl^ Myo15-cre^+/–^* KO (P24 [**B**]; P25 [**D**]) mice during the extracellular perfusion of 40 mM KCl (holding potential: –84 mV). The size of the slow-activating and sustained inward current, which is independent from the efferent system activation, varied among cells and was larger in *Myo7a^fl/fl^ Myo15-cre^+/–^* (365 ± 230 pA, *n* = 129) compared with *Myo7a^fl/fl^* mice (165 ± 153 pA, *n* = 108) due to the smaller K^+^ currents active at the holding potential of –84 mV (15). Note that the superimposed inhibitory synaptic currents (IPSCs) were only evoked in *Myo7a^fl/fl^ Myo15-cre^+/–^* (see also expanded view in **D**), which were blocked by the selective α9α10nAChR blocker strychnine. (**E**) Percentage of IHCs responding to 40 mM KCl with IPSCs as a function of age. Numbers above the data represent the IHCs showing IPSCs versus total IHCs tested. (**F** and **G**) Average frequency (**F**) and amplitude (**G**) of the IPSCs as a function of age. Number of mice in **E**–**G** for each age group from left to right are: *Myo7a^fl/fl^*: *n* = 7, 10, 14, 26, 17; *Myo7a^fl/fl^ Myo15-cre^+/–^*: *n* = 7, 7, 9, 16, 17. Data are shown as mean ± SD.

Considering that the ACh-activated current can be elicited in the IHCs from *Myo7a^fl/fl^ Myo15-cre^+/–^* at P22 (15), and SK2 channels are significantly upregulated in IHCs after P22 (Figure 2), we investigated when the axo-somatic efferent contacts on the IHCs of *Myo7a^fl/fl^ Myo15-cre^+/–^* mice became active. The presence of functional axo-somatic efferent contacts was tested by applying an extracellular solution containing 40 mM KCl, instead of the normal 5.8 mM, onto the cochlear epithelium. High-K^+^ caused IHCs, which were voltage clamped at –84 mV, to respond with an inward sustained current owing to a positive shift in the K^+^ reversal potential from –81 mV (5.8 mM K^+^) to –31 mV (40 mM K^+^). In addition, high-K^+^ also depolarizes the efferent synaptic terminals causing the release of ACh-containing vesicles and the generation of transient inhibitory postsynaptic currents (IPSCs) superimposed on the KCl-induced sustained inward current in IHCs (21, 29). We found that, in *Myo7a^fl/fl^ Myo15-cre^+/–^* mice, 40 mM KCl elicited robust and reliable efferent synaptic currents in the large majority of IHCs starting from P25 (Figure 3, A–E). These currents were blocked by 1M strychnine (Figure 3D), a potent blocker of α9α10nAChRs (30), confirming the reexpression of these receptors in adult *Myo7a^fl/fl^ Myo15*-cre^+/–^ IHCs. In *Myo7a^fl/fl^ Myo15*-cre^+/–^ mice, we found that both the frequency and amplitude of the IPSCs were similar across the different age ranges (*P* = 0.5890, *P* = 0.4089, respectively; 1-way ANOVA; Figure 3, F and G). Both the frequency (4.8 ± 3.2 Hz, range 0.5–14.1 Hz, P24–P42) and amplitude (76 ± 46 pA, range 27–317 pA, P24–P42) of IPSCs from all 70 IHCs were comparable with those previous reported (15, 29). These results indicate that knocking out *Myo7a* causes the presynaptic efferent terminals to innervate the IHCs, or become functional, only a few days after the acquisition of the postsynaptic elements in the IHCs.

### Afferent ribbon synapses are retained during reinnervation of IHCs by the efferent system.

**Figure 4:**
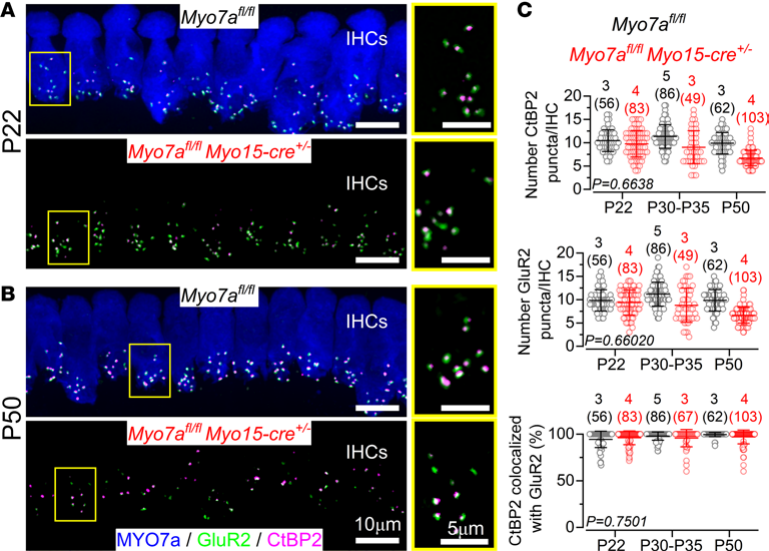
Ribbon synapse number is not affected in Myo7a^fl/fl^ Myo15-cre^+/–^ mice. (**A** and **B**) Maximum intensity projections of confocal *Z* stack images taken from the 9–12 kHz cochlear region in *Myo7a^fl/fl^* and *Myo7a^fl/fl^ Myo15-cre^+/–^* mice at P22 (**A**) and P50 (**B**). IHCs were labeled with antibodies against the ribbon synapse marker CtBP2 (magenta), the postsynaptic marker GluR2 (green), and the cell marker MYO7A (blue). The right panels in **A** and **B** show a higher magnification of the synaptic region within the boxed IHCs depicted in the left panels. Colocalization between the pre- (CtBP2) and postsynaptic (GluR2) markers is highlighted in white. Scale bars: 10 μm (left), 5 μm (right).(**C**) Number of CtBP2 puncta (top panel) and GluR2 puncta (middle panel) per IHC; bottom panel shows the percentage of colocalized CtBP2-GluR2 puncta, at each age group tested in *Myo7a^fl/fl^* (black) and *Myo7a^fl/fl^ Myo15-cre^+/–^* (red) mice. Significance was found using 2-way ANOVA. Data are shown as mean ± SD. The number of mice is indicated above the data groups and numbers of IHCs is shown in parentheses.

In the adult cochlea, the LOC efferent neurons form axo-dendritic synapses with the SGN terminals that contact the IHCs, the majority of which being predominantly located on the modiolar side of the cell (31). Considering that the modiolar SGNs are believed to be more vulnerable to damage and are largely reduced during aging (32, 33), it has been suggested that the disappearance of their physiological target could prompt the unconnected efferent terminals to establish direct axo-somatic contacts with IHCs (24, 26, 34). Therefore, we tested this hypothesis in *Myo7a^fl/fl^ Myo15-cre^+/–^* mice by evaluating the number of afferent synapses using antibodies against the presynaptic ribbon protein RIBEYE (CtBP2) and the postsynaptic AMPA-type glutamate receptor GluR2 (32, 35). We found that both CtBP2 and GluR2 puncta were present in the pre- and postsynaptic sites at each age tested (P22–P50) in both control and *Myo7a^fl/fl^ Myo15-cre^+/–^* mice (Figure 4, A–C). The number of CtBP2 and GluR2 puncta, as well as the number of colocalized puncta (indicative of functional afferent synapses) in 150 μm of the apical cochlear region (9–12 kHz), did not significantly differ between both genotypes over the age range investigated (CtPB2, *P* = 0.6638; GluR2, *P* = 0.6020; percentage of colocalization, *P* = 0.7501, 2-way ANOVA; Figure 4C). Overall, these data indicate that the reestablishment of the axo-somatic efferent synapse in *Myo7a^fl/fl^ Myo15-cre^+/–^* mice, which occurs as early as P24, was not due to the loss of afferent fibers.

### Knockdown of Myo7a in IHCs alone is sufficient to induce efferent reinnervation.

**Figure 5:**
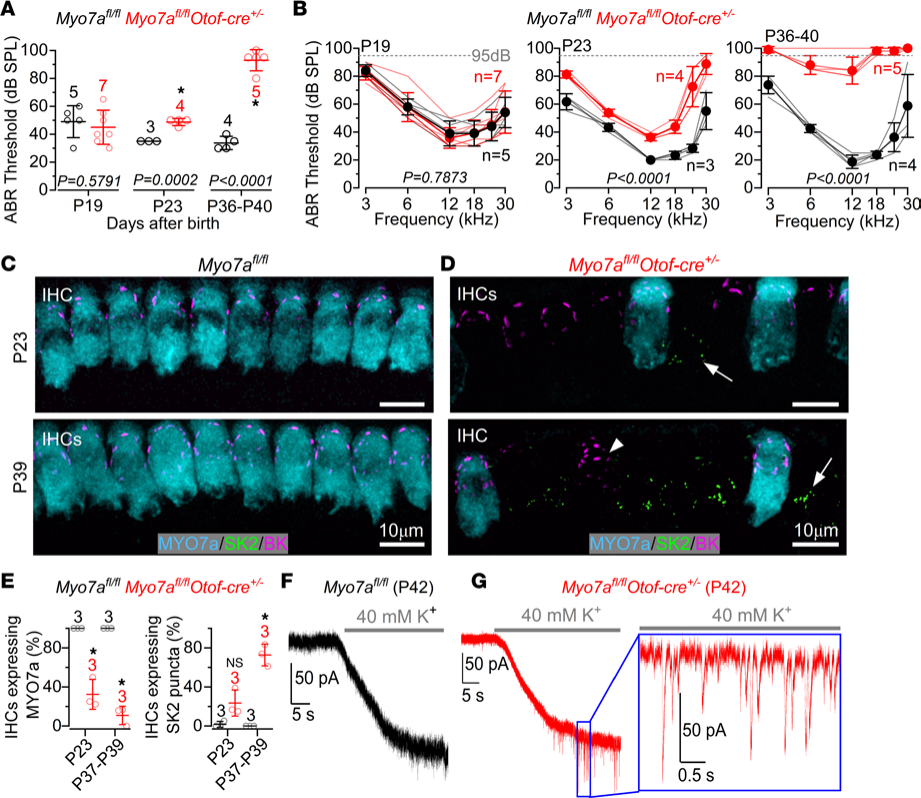
Conditional KO of Myo7a only in the IHCs is sufficient to induce the efferent reinnervation. (**A**) Average ABR thresholds for click stimuli recorded from *Myo7a^fl/fl^* (black) and *Myo7a^fl/fl^ Otof-cre^+/–^* (red) mice at P19, P23, and P36–P40. Two-tailed *t* test was used. (**B**) ABR thresholds for frequency-specific pure tone burst stimuli from 3 to 30 kHz recorded from both genotypes at P19 (left), P23 (middle), and P36–P40 (right). Significance was found using 2-way ANOVA. Number of mice tested for each genotype is shown next to the data. The dashed lines indicate the upper threshold limit for these recordings (95 dB). (**C** and **D**) Maximum intensity projections of confocal *Z* stack images taken from the 9–12 kHz apical region of the cochlea in *Myo7a^fl/fl^* (**C**) and *Myo7a^fl/fl^ Otof-cre^+/–^* mice (**D**) at P23 (top panels) and P39 (bottom panels). IHCs were labeled with antibodies against SK2 (green), BK (magenta), and the IHC marker MYO7A (cyan). BK channels are expressed in mature IHCs. Arrows point to SK2 puncta; arrowhead indicates BK puncta. Scale bars: 10 μm. (**E**) Percentage of IHCs that expressed MYO7A (left) and SK2 (right) puncta in 150 μm of the apical cochlea region at P19 or P37–P39 in *Myo7a^fl/fl^* (black) and *Myo7a^fl/fl^ Otof-cre^+/–^* mice (red). *P < 0.0001. NS indicates *P* = 0.0645 (Tukey’s post hoc test, 1-way ANOVA). The number of mice used for each genotype is shown above the data. (**F** and **G**) Voltage-clamp recordings obtained from IHCs in *Myo7a^fl/fl^* (**F**) and *Myo7a^fl/fl^ Otof-cre^+/–^* (**G**) mice at P42 during the extracellular application of 40 mM KCl. IPSCs were only evoked in *Myo7a^fl/fl^ Otof-cre^+/–^* IHCs. Recordings were made from 3 *Myo7a^fl/fl^* and 4 *Myo7a^fl/fl^ Otof-cre^+/–^* mice. Data are shown as mean ± SD.

Having established that the efferent reinnervation of IHCs in *Myo7a^fl/fl^ Myo15-cre^+/–^* mice was not associated with a loss of SGNs, we tested whether it was a secondary effect caused by lack of MYO7A in the outer hair cells (OHCs). This is because adult OHCs, which are innervated by MOC efferent fibers, are known to be more susceptible than IHCs to cochlear insult, such as loud noise or aging (36, 37). If OHCs are lost or degenerate, then the unconnected MOC fibers could divert toward the IHCs. To address this hypothesis, we crossed the *Myo7a^fl/fl^* with *Otof*-cre mice (Figure 5, A–G), which express cre-recombinase under the otoferlin promoter that primarily targets IHCs (38, 39). Similar to *Myo7a^fl/fl^ Myo15-cre^+/–^*, we found that *Myo7a^fl/fl^ Otof-cre^+/–^* mice had normal hearing thresholds at least up to P19 but rapidly increased with age such that, by P40, they were almost completely deaf (Figure 5, A and B). We verified the overall function of OHCs from *Otof*-cre mice by measuring distortion product otoacoustic emissions (DPOAEs), which are a product of cochlear amplification caused by OHC electromotility during acoustic stimulation of their hair bundles. We found that DPOAE thresholds were indistinguishable between the 2 genotypes at P39–P44 (Supplemental Figure 4), indicating that OHCs were functional. Although *Myo7a^fl/fl^ Otof-cre^+/–^* mice do not downregulate MYO7A in 100% of the IHCs (Figure 5, C–E), they offer the advantage of allowing comparisons within the same cochlea between cells with and without MYO7A. A characteristic of adult IHCs is the expression of large conductance calcium-activated potassium channels (BK) at their neck region, which carry the rapid-activating outward K^+^ current *I*_K,f_ (40, 41). We found that IHCs from *Myo7a^fl/fl^ Otof-cre^+/–^* mice that still expressed the BK channels did not show SK2 channels, which are normally present only in immature IHCs (22, 23). However, several IHCs from *Myo7a^fl/fl^ Otof-cre^+/–^* mice lacked BK channels and instead reexpressed SK2 channels (Figure 5, C–E), which is a sign of the reestablishment of the efferent postsynaptic specialization. The presence of functional axo-somatic efferent synapses in *Myo7a^fl/fl^ Otof-cre^+/–^* mice was supported by the presence of synaptic currents in response to the application of 40 mM extracellular KCl (Figure 5, F and G). IPSCs recorded from IHCs of P42–P50 *Myo7a^fl/fl^ Otof-cre^+/–^* mice had an average frequency of 2.3 ± 1.2 Hz (range 1.2–4.3.1 Hz, 7 IHCs, 4 mice) and amplitude of 47 ± 12 pA (range 32–71 pA). These values were not significantly different from those measured in *Myo7a^fl/fl^ Myo15-cre^+/–^* mice of a comparable age range (P36–P42 from Figure 3, F and G: 3.9 ± 3.5 Hz, *P* = 0.4744; 71 ± 33 pA, *P* = 0.0606; 25 IHCs; Mann-Whitney *U* test). Overall, the above findings indicate that the targeted deletion of *Myo7a* in IHCs alone is sufficient to reestablish axo-somatic efferent reinnervation in the adult cochlea.

### Disruption of IHC exocytosis does not lead to efferent reinnervation.

**Figure 6:**
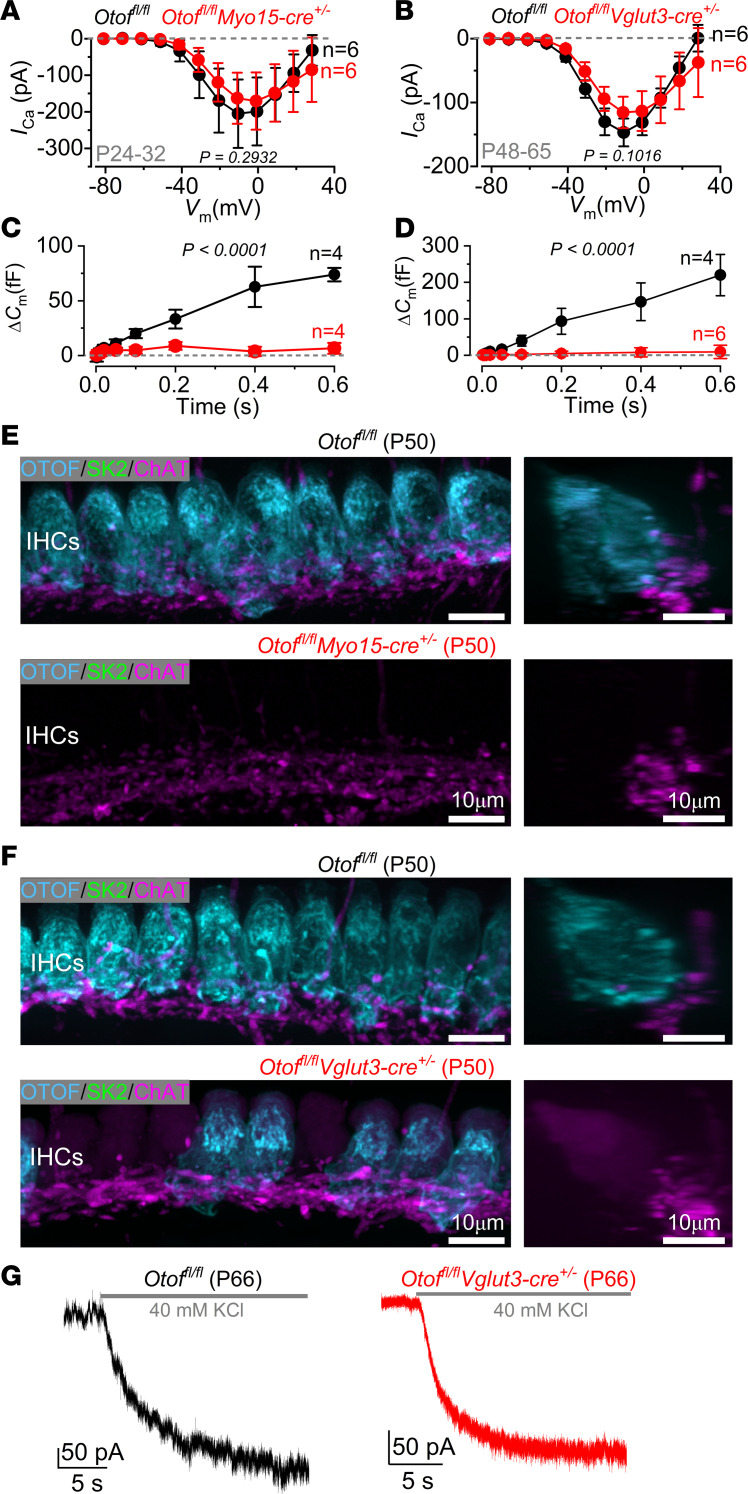
Abolishing IHC exocytosis does not trigger efferent reinnervation. (**A**) Average peak Ca^2+^ current-voltage (*I_Ca_*-*V*_m_) curves from control *Otof^fl/fl^* (black) and *Otof^fl/fl^ Myo15-cre^+/–^* (red) mice between P24 and P32. Recordings were obtained in response to 50 ms voltage steps from –81 mV in 10 mV increments. (**B**) *I_Ca_*-*V*_m_ curves from control *Otof^fl/fl^* and *Otof^fl/fl^Vglut3-cre^+/–^* mice between P48 and P65. Recording conditions are as in **A**. (**C** and **D**) Exocytosis was recorded from both *Otof^fl/fl^ Myo15-cre^+/–^* (**C**) and *Otof^fl/fl^Vglut3-cre^+/–^* (**D**) mice and their respective controls (*Otof^fl/fl^*) mice from the same age-range stated in **A** and **B**, respectively. Δ*C*_m_ was elicited by applying 50 ms voltage steps to –11 mV (holding potential: –81 mV) between 2 ms and 0.6 seconds (interstep interval: at least 11 seconds) using 1.3 mM extracellular Ca^2+^ and at 35°C–37°C. Data are shown as mean ± SD. Significance was found using 2-way ANOVA. Number of IHCs is shown next to the data. Number of mice: *Otof^fl/fl^* and *Otof^fl/fl^ Myo15-cre^+/–^* (*n* = 5 [**A**], 4 [**C**]); *Otof^fl/fl^* (*n* = 3 [**B**], 2 [**D**]) and *Otof^fl/fl^Vglut3-cre^+/–^* (*n* = 3 [**B**], 3 [**D**]). (**E** and **F**) Maximum intensity projections of confocal *Z* stacks of IHCs taken from the apical cochlea region of *Otof^fl/fl^* and *Otof^fl/fl^ Myo15-cre^+/–^* (**E**) and *Otof^fl/fl^Vglut3-cre^+/–^* (**F**) mice at P50. IHCs were labeled with antibodies against the efferent presynaptic terminal marker ChAT (magenta), the postsynaptic efferent marker SK2 (green), and otoferlin (cyan). Right images show a side view of an IHC from the left images. SK2 puncta were absent in the IHCs of both strains. Three mice were used for each genotype and age group. Scale bars: 10 μm. (**G**) Voltage-clamp recordings obtained from IHCs held at –84 mV in P66 *Otof^fl/fl^* and *Otof^fl/fl^Vglut3-cre^+/–^* mice during the extracellular application of 40 mM KCl. Different from *Myo7a^fl/fl^ Myo15-cre^+/–^* mice (Figure 3), IHCs responded to KCl with slow inward sustained currents (*Otof^fl/fl^*: 343 ± 126 pA, 3 IHCs from 2 mice; *Otof^fl/fl^Vglut3-cre^+/–^*: 355 ± 98 pA, 5 IHCs from 4 mice; *P* = 0.8745, 2-tailed *t* test) without the superimposed IPSCs in both genotypes.

Similar to our findings in *Myo7a*-deficient mice, the efferent reinnervation of IHCs in the aged cochlea occurs at a time when the MET current is reduced compared with that of young adult mice (25). A reduction in the IHC MET current could decrease or even abolish the activity of some of the afferent terminals, even if they are still physically intact. The lack of activity in the target afferent fiber could trigger the LOC fibers to rewire onto the IHCs. To selectively silence the activity of the afferent fibers, we used 2 conditional KO mice to delete otoferlin in the IHCs (Figure 6), which is crucial for exocytosis at their ribbon synapses (42). This was achieved by crossing *Otof^fl/fl^* mice with either *Myo15-cre* or the tamoxifen inducible *Vglut3-cre* (43). The vesicular glutamate transporter 3 (VGLU3) is essential for glutamate release at IHC ribbon synapse since it is required to transport and repackage glutamate into synaptic vesicles (44, 45). While otoferlin expression was completely absent in the IHCs of *Otof^fl/fl^ Myo15-cre^+/–^* mice at P16 (Supplemental Figure 5), that in *Otof^fl/fl^Vglut3-cre^+/–^* was only reduced by 35% ± 4 % (*n* = 4) at 4 weeks after the injection of tamoxifen. However, the latter strain allowed us to make side-by-side comparisons of IHCs with and without otoferlin.

We first established whether exocytosis in the IHCs from the above mouse lines was abolished (Figure 6, A–D). IHC exocytosis was estimated by measuring increases in cell membrane capacitance (*C*_m_) following depolarizing voltage steps that activate the calcium current (*I_Ca_*). *C*_m_ is generally interpreted as a sign of neurotransmitter release from presynaptic cells (46–48). We found that the size of *I_Ca_*, which was elicited by applying voltage steps in 10 mV increments from –81 mV to more depolarized potentials, was not significantly different between controls *Otof^fl/fl^* and either of the KO mice (*Otof^fl/fl^ Myo15-cre^+/–^*, *P* = 0.2932; *Otof^fl/fl^Vglut3-cre^+/–^*, *P* = 0.101; 2-way ANOVA; Figure 6, A and B, respectively). However, the rate of neurotransmitter release in IHCs, which was studied by measuring *C*_m_ at the peak *I_Ca_* (–11 mV) in response to depolarizing voltage steps from 2 ms to 0.6 seconds in duration, was almost completely abolished in both *Otof-*deficient mice (*P* < 0.0001 for both comparisons, 2-way ANOVA; Figure 6, C and D). Despite the absence of exocytosis in both conditional *Otof*-deficient mice, IHCs did not express SK2 channels (Figure 6, E and F) nor did they show any efferent-mediated synaptic currents (Figure 6G), indicating that abolishing exocytosis and thus the afferent activity did not trigger efferent reinnervation of IHCs in the adult cochlea.

### Nature of the newly formed efferent synapses in IHCs lacking MYO7A.

Recent data from aged mice have suggested that LOC fibers are the most likely candidate to reinnervate the IHCs (25). To confirm this observation, we performed immunostaining experiments using the anti-ATP1A3 antibody, which labels the MOC efferent neurons (49) known to contact the OHCs in the adult cochlea (19, 50). Since the anti-ATP1A3 antibody has also been shown to label the afferent fibers, MOC fibers are identified as those that also showed expression of the efferent marker ChAT. Using this approach, we confirmed that the efferent synapses on the OHCs from both *Myo7a^fl/fl^* and *Myo7a^fl/fl^ Myo15-cre^+/–^* mice were MOC fibers since they showed both ATP1A3 and ChAT labeling (Supplemental Figure 6, A–F). However, the efferent synapses on IHCs from *Myo7a^fl/fl^ Myo15-cre^+/–^* mice were not labeled by the ATP1A3 antibody (Supplemental Figure 6F), suggesting they were LOC terminals. We also found that in IHCs, ChAT^+^ terminals were found juxtaposed to the efferent postsynaptic SK2 channel puncta only in *Myo7a^fl/fl^ Myo15-cre^+/–^* mice (Supplemental Figure 6, G and H), further supporting the reexpression of the postsynaptic machinery.

### AAV-mediated Myo7a rescue in posthearing mice partially restores IHC and hearing function in Myo7a-deficient mice.

We next assessed the plasticity of the adult sensory epithelium by testing whether the AAV-mediated in vivo replacement of *Myo7a* in posthearing mice could restore the function of the MET apparatus and revert the efferent innervation back to its normal adult-like configuration. To do this, we used a dual AAV approach to deliver Myo7a cDNA into the cochlear perilymphatic space in vivo via the round window membrane (RWM) of P13–P15 control and *Myo7a*-deficient mice.

**Figure 7:**
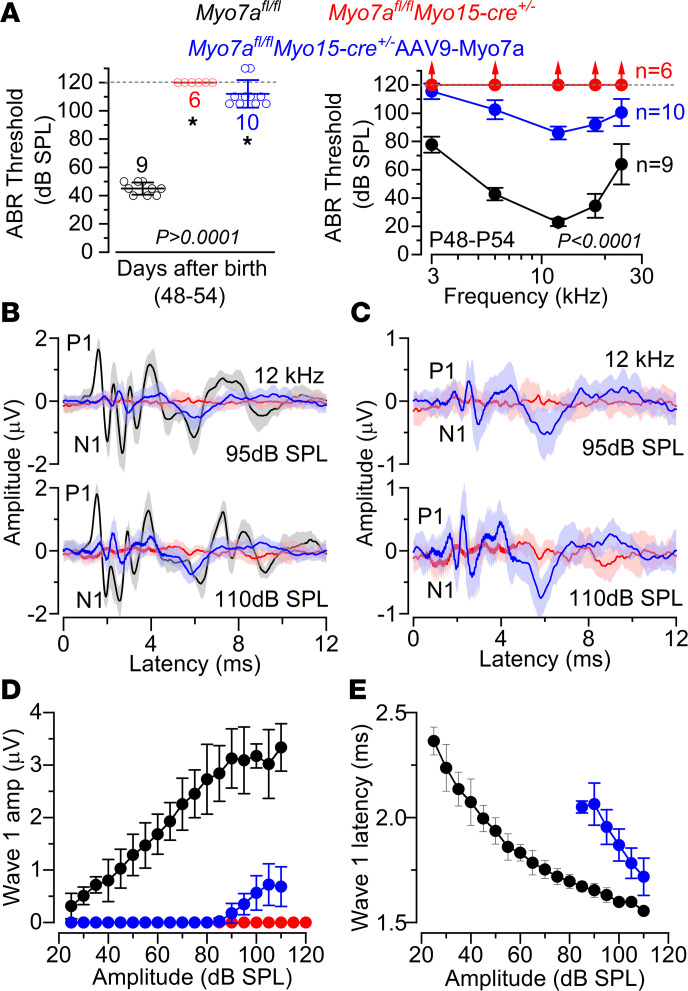
Auditory brainstem response thresholds partially recover in Myo7a^fl/fl^ Myo15-cre^+/–^ mice injected with AAV9-Myo7a. (**A**) Average ABR thresholds for click (left) and frequency-specific pure tone burst (right) stimuli recorded from *Myo7a^fl/fl^* (black), *Myo7a^fl/fl^ Myo15-cre^+/–^* (red), and *Myo7a^fl/fl^ Myo15-cre^+/–^*AAV9*-Myo7a* (blue) mice at P48–P54. The number of mice used is shown near the data points. The dashed lines indicate the upper threshold limit used for this experiment (120 dB SPL). For click thresholds, statistical comparison is from 1-way ANOVA (Tukey’s post hoc test: *P* < 0.0001 between control and *Myo7a^fl/fl^ Myo15-cre^+/–^* without or with surgery; *P* = 0.0001 between *Myo7a^fl/fl^ Myo15-cre^+/–^* without and with surgery). For pure tone thresholds, statistical comparison is from 2-way ANOVA (Tukey’s post hoc test: *P* < 0.0001 for all comparisons). (**B**) Average ABR waveform responses at 12 kHz and using 2 stimulus intensities (95 and 110 dB SPL) relative to threshold from the 3 different mouse lines described in **A**. Continuous lines represent mean values, and shaded areas represent the SD. P1 and N1 indicate the positive and negative peaks of wave 1. (**C**) Same average ABR waveform responses as shown in **B** but only for the noninjected *Myo7a^fl/fl^ Myo15-cre^+/–^* and *Myo7a^fl/fl^ Myo15-cre^+/–^*AAV9*-Myo7a* mice to better highlight the size of wave 1. (**D** and **E**) Average amplitude (**D**) and latency (**E**) of wave 1 (from P1 to N1) for 3 conditions described in **A**. Please note that, due to wave 1 amplitude being zero in *Myo7a^fl/fl^ Myo15-cre^+/–^* mice (red), the latency could not be measured in **E**. Data in **A**, **B**, **D**, and **E** are shown as mean ± SD.

The hearing function of the treated mice was assessed using ABRs. We found that for click ABR stimuli, noninjected WT mice had thresholds around 45 dB sound pressure level (SPL), while noninjected *Myo7a^fl/fl^ Myo15-cre^+/–^* mice had no detectable thresholds even at levels up to 120 dB SPL (Figure 7A). Most AAV9*-Myo7a*–injected *Myo7a^fl/fl^ Myo15-cre^+/–^* mice had significantly lower thresholds compared with noninjected *Myo7a^fl/fl^ Myo15-cre^+/–^* mice, although their thresholds remained significantly elevated compared with controls (*P* < 0.0001, Tukey’s post hoc test, 1-way ANOVA; Figure 7A). For pure tone ABRs, we found a similar trend as for the clicks, with an average recovery of about 30 dB across all frequencies tested in the AAV-injected mice (between 3 kHz and 24 kHz) (Figure 7A). We then analyzed the amplitude and latency of the ABR wave 1 at 12 kHz and at 2 sound intensities (95 and 110 dB SPL). We found that the amplitude and latency of wave 1 was reemerging in *Myo7a^fl/fl^ Myo15-cre^+/–^* mice transduced with AAV9*-Myo7a* for sound intensities above 90 dB (Figure 7, B–E). These results are encouraging considering that dual-AAV9-*Myo7a* does not transduce 100% of adult IHCs (Supplemental Figure 7). Injection into the cochlea of either AAV9-*Myo7A*-Nterm or AAV9-*Myo7a*-Cterm alone failed to produce MYO7A in hair cells (Supplemental Figure 8).

**Figure 8:**
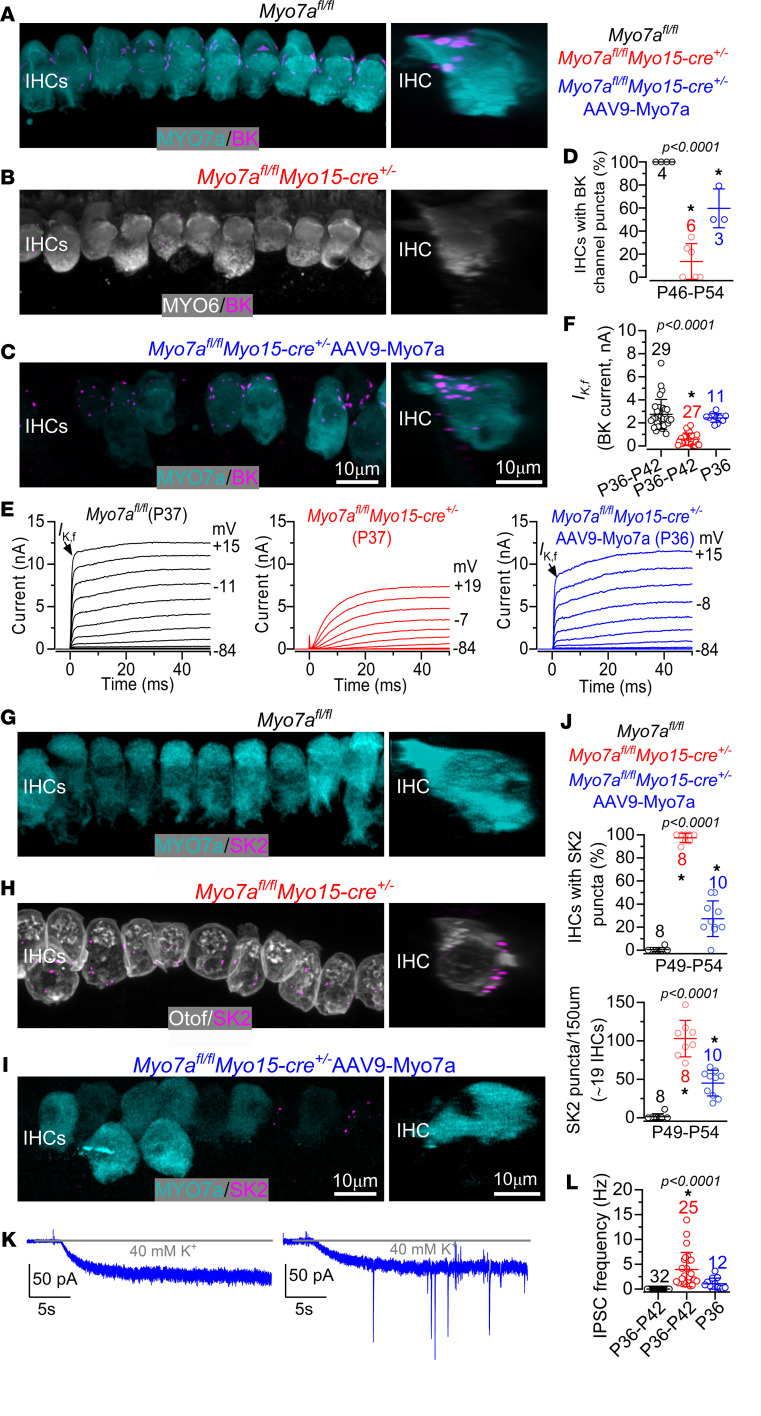
Injection of AAV9-Myo7a rescues the normal efferent wiring of the adult IHCs in Myo7a^fl/fl^ Myo15-cre^+/–^ mice. (**A**–**C**) Maximum intensity projections of confocal *Z* stack images taken from the 9–12 kHz cochlear region in noninjected control *Myo7a^fl/fl^* (**A**), *Myo7a^fl/fl^ Myo15-cre^+/–^* (**B**), and *Myo7a^fl/fl^ Myo15-cre^+/–^* mice injected with AAV9*-Myo7a* (**C**) at P49–P54. AAV-*Myo7a* was injected between P13 and P15. Cochleae were labeled with antibodies against BK (magenta) and the IHC markers MYO7A (cyan) or MYO6 (gray). BK was almost complete absence in *Myo7a^fl/fl^ Myo15-cre^+/–^* mice (**B**). Right images show a single IHC from the left panels rotated on the *y*,*z* plane, providing a lateral view of the IHC and allowing visualization of the juxtaposed MYO7A/MYO6 and the BK puncta. (**D**) Percentage of IHCs expressing the BK channels over 150 μm range. Statistical comparisons (post hoc test, 1-way ANOVA): *Myo7a^fl/fl^ vs.* *Myo7a^fl/fl^ Myo15-cre^+/–^*, *P* < 0.0001; *Myo7a^fl/fl^ vs.* *Myo7a^fl/fl^ Myo15-cre^+/–^*AAV9-Myo7a, *P* = 0.0068; *Myo7a^fl/fl^ Myo15-cre^+/–^* *vs.* *Myo7a^fl/fl^ Myo15-cre^+/–^*AAV9-Myo7a, *P* = 0.0016. Number of mice for **A**–**C** is shown in **D**. (**E**) Example of outward K^+^ current responses from IHCs of P37 *Myo7a^fl/fl^*, P37 *Myo7a^fl/fl^ Myo15-cre^+/–^* and P36 *Myo7a^fl/fl^ Myo15-cre^+/–^* mice injected with AAV9*-Myo7a*. Currents were elicited by using 10 mV depolarizing voltage steps from –84 mV to the various test potentials shown by some of the traces. BK current *I*_K,f_ is indicated with an arrow. (**F**) Size of the isolated *I*_K,f_ measured at –25 mV and at 1 ms from the onset in the 3 experimental conditions shown in **E**. The number of IHCs is shown above the data. Number of mice from left to right: 15, 16, 3. (**G**–**I**) Images obtained as described in **A**–**C** for the same 3 mouse lines (P49–P54) and using antibodies against SK2 (magenta) and the IHC marker MYO7A (cyan) and otoferlin (gray). (**J**) Percentage of IHCs expressing the SK2 channels and number of SK2 puncta over 150 μm of the apical cochlear region. Number of mice used in **G**–**I** is shown in **J**. (**K**) Examples of inward membrane currents recorded from IHCs of P36 *Myo7a^fl/fl^ Myo15-cre^+/–^* mice injected with AAV9*-Myo7a*. Recording protocol is as described in Figure 3. Note that 1 IHC (left) only shows the inward current, while in the others (right) 40 mM K^+^ also elicited a few IPSCs. (**L**) Average frequency of the IPSCs recorded from IHCs of 17 *Myo7a^fl/fl^,* 17 *Myo7a^fl/fl^ Myo15-cre^+/–^* and 3 *Myo7a^fl/fl^ Myo15-cre^+/–^* mice injected with AAV9*-Myo7a*. One-way ANOVA was used. Data are shown as mean ± SD.

As mentioned above (Figure 5, C and D), BK channels are a distinctive characteristic of adult IHCs and are expressed around their neck region (Figure 8, A and D). While IHCs from *Myo7a^fl/fl^ Myo15-cre^+/–^* mice were almost completely devoid of BK channels (Figure 8, B and D), those from *Myo7a^fl/fl^ Myo15-cre^+/–^* mice transduced with AAV9-*Myo7a* reexpress BK channels (Figure 8, C and D). The reexpression of BK channels in the IHCs of *Myo7a^fl/fl^ Myo15-cre^+/–^* mice transduced with AAV9-*Myo7a* was confirmed electrophysiologically, since the size of *I*_K,f_ was significantly upregulated compared with that recorded in the IHCs of *Myo7a^fl/fl^ Myo15-cre^+/–^* mice (*P* < 0.0001, Tukey’s post hoc test, 1-way ANOVA) but comparable with that of control *Myo7a^fl/fl^* cells (*P* = 0.6246) (Figure 8, E and F). The presence of a large *I*_K,f_ was not due to the injection of AAV9-*Myo7a* in P13 mice preventing the observed downregulation of BK channels in the IHCs of *Myo7a^fl/fl^ Myo15-cre^+/–^* mice (15), since *I*_K,f_ was still very small or absent at P27 (Supplemental Figure 9, A and B). We then investigated whether adult IHCs transduced with AAV9-*Myo7a* returned to their normal functional state by also downregulating the efferent postsynaptic SK2 channels and their ability to respond to the efferent system (Figure 8, G–L). We found that IHCs from *Myo7a^fl/fl^ Myo15-cre^+/–^* mice exhibited a robust reexpression of SK2 channels (Figure 8, H and J), as described in Figure 2. However, SK2 puncta were almost completely absent in IHCs transduced with AAV9-*Myo7a* (Figure 8, I and J), which is similar to control IHCs (Figure 8, G and J). We also found that the extracellular application of 40 mM KCl onto the IHCs of *Myo7a^fl/fl^ Myo15-cre^+/–^* mice transduced with AAV9-*Myo7a* elicited inward currents with no or very few superimposed IPSCs, the frequency of which was significantly reduced compared with that recorded in IHCs from *Myo7a^fl/fl^ Myo15-cre^+/–^* mice (*P* = 0.0009, Tukey’s post hoc test, 1-way ANOVA). However, IHC responses to high-K^+^ were not significantly different from those recorded from control mice (*P* = 0.2918). As for the BK channels (Supplemental Figure 9, A and B), the downregulation of the efferent activity was not due to the transduction of AAV9-*Myo7a* at P13 preventing the normal changes seen in IHCs from *Myo7a^fl/fl^ Myo15-cre^+/–^* mice (Figure 2 and Figure 3), since SK2 channels and IPSCs were present in P22–P27 IHCs (Supplemental Figure 9, C–F).

The functional recovery of *Myo7a*-deficient IHCs following the transduction of AAV9-*Myo7a* was further validated on *Myo7a^fl/fl^ Otof-cre^+/–^* mice (Supplemental Figure 10, A–G). The in vivo delivery of AAV9-*Myo7a* to P13 *Myo7a^fl/fl^ Otof-cre^+/–^* mice led to the partial recovery of ABR thresholds (*P* < 0.0001, 2-way ANOVA; Supplemental Figure 10A). As shown for *Myo7a^fl/fl^ Myo15-cre^+^*, IHCs from *Myo7a^fl/fl^ Otof-cre^+/–^* mice downregulate the BK channels, reexpress SK2 channels, and show IPSC responses to efferent activation (Figure 5). However, as seen for *Myo7a^fl/fl^ Myo15-cre^+/–^* mice (Figure 8), IHCs from *Myo7a^fl/fl^ Otof-cre^+/–^* mice transduced with AAV9-*Myo7a* reexpressed BK channels (Supplemental Figure 10, B and C) and no longer showed IPSCs (Supplemental Figure 10, D–G).

The above results indicate that the reestablishment of MYO7A expression, and thus partial ABR recovery, was sufficient for the IHCs to establish MET current, downregulate the efferent postsynaptic machinery, and no longer respond to efferent stimulation.

## Discussion

In this study, we demonstrate that the reestablishment of axosomatic contact between the cholinergic efferent system and the IHCs in the adult cochlea is caused by defective mechanoelectrical transduction. This abnormal efferent innervation, which is also a major feature observed in the cochlea of aged mice (24–26), can be reversed in vivo using AAV-based gene rescue therapy. During efferent rewiring, the postsynaptic α9α10nAChRs are reexpressed in the IHCs (15) a couple of days prior to the appearance of SK2 channels and efferent-driven synaptic currents, mimicking efferent synaptogenesis during early stages of prehearing development (28). We also show that efferent reinnervation of adult IHCs was not an indirect effect caused by the loss of OHCs or afferent fibers, nor is it due to the silencing of IHCs by the prevention of exocytosis, which have all been previously suggested reasons for the formation of these de novo synapses in the aged cochlea. The exogenous delivery of the deafness gene (*Myo7a*) via dual-AAV into *Myo7a-*deficient IHCs reestablished the normal protein expression profile of adult IHCs and partially restores hearing. These findings also indicate that the adult cochlea is more “plastic” than previously thought and highlight the potential of using gene therapy for progressive hearing loss.

### Trigger of the efferent-IHC axosomatic innervation in the adult cochlea.

In the prehearing cochlea, the MOC efferent endings make transient axosomatic synaptic contacts with IHCs (19, 51), the role of which is to modulate the frequency of Ca^2+^-dependent action potentials that are transiently present in immature IHCs (21, 29, 52). Preventing this modulation from the efferent system on the developing IHCs has been shown to affect the normal maturation of IHC ribbon synapses (29) and the refinement of tonotopic maps in the maturing brainstem (53). Adult IHCs no longer respond to ACh (21), highlighting the specific role of the efferent system in regulating the development of the auditory system. Although the MOC efferent system is still present in the adult cochlea, it directly suppresses OHC electromotility — and thus cochlear amplification — to prevent, for example, noise-induced damage (16). While the activity of adult IHCs is no longer modulated by the MOC fibers, the LOC efferent fibers form axodendritic synapses with the SGN afferent terminals that contact the IHCs (19), the function of which is less well understood due to the multiple neurotransmitters known to be secreted by these synapses (16). Although the reestablishment of the direct efferent-IHC synapses has previously been described in the adult and aged cochlea (15, 24–26), and following the presentation of noxious stimuli such as loud noise and aminoglycoside treatment (54, 55), the trigger for this efferent plasticity has remained elusive.

In the aging cochlea, the reemergence of direct efferent-IHC synapses occurs simultaneously with several additional cochlear pathologies such as the loss of OHCs and afferent fibers. This has led to the hypothesis that a loss of synaptic target may drive the unconnected MOC or LOC terminals to contact the surviving IHCs (16, 26, 56). However, we have shown that the reemergence of the efferent-IHC axosomatic terminals was not driven by the loss of either OHCs or afferent fibers, nor was it prevented by completely “silencing” the IHCs via abolishing exocytosis. Instead, we demonstrate that the trigger for efferent rewiring is directly linked to the dysfunctional MET current. Defects in mechanoelectrical transduction have also been reported in aged IHCs (25) that show disrupted hair bundles (57, 58), following noise exposure (59, 60) and after aminoglycoside treatment (61), all of which leading to efferent rewiring of the IHCs. It is likely that the reduced MET current flowing into the IHCs at rest and/or during acoustic stimulation leads to membrane hyperpolarization, which potentially affects gene expression and, consequently, the maintenance of the adult IHC biophysical profile and innervation pattern (62, 63).

### Synaptogenesis of the efferent-IHC innervation in the adult cochlea.

The progression of reestablishing the direct LOC-IHC innervation in adult mice is strikingly similar to the transient MOC-IHC axosomatic synapses established during development (28). In newborn mice, nAChRs are the first proteins to appear in the IHC efferent synaptic complex, which is followed by the expression of SK2 channels about a day later. The outgrowing efferent axons have been shown to make initial contacts with the IHCs from just after birth (64–66); this occurs at a time when the postsynaptic nAChRs are already present and functional but still uncoupled from SK2 channels (28). This led to the hypothesis that ACh release from the outgrowing efferent axons could lead to Ca^2+^ influx into the IHCs via the activation of nAChRs and therefore provide an instructive signal for synaptogenesis (28). A similar mechanism could also underlie the efferent reinnervation of adult IHCs with a dysfunctional MET current or normal IHCs in aged animals. We found that LOC fibers are the most likely candidate reinnervating the IHCs deprived of functional MET current, similar to the LOC efferent reinnervation seen in aged mice (25). The efferent fibers of the inner ear have a common embryological origin as facial branchial motor neurons, which operate via cholinergic synapses at their neuromuscular junctions (67). At the neuromuscular junction, aging is associated with significant synapse remodeling, including an increase in the presynaptic terminal branching, and has been interpreted as an attempt to improve communication between pre- and postsynaptic components (68). A similar terminal branching mechanism could account for the additional LOC efferent fibers that reform synaptic contacts with the IHCs during aging and in adult mice with dysfunctional MET current. Interestingly, recent studies on the neuromuscular junction have also highlighted that postsynaptic efferent differentiation could occur in the absence of efferent terminals, indicating the presence of a prepatterned postsynaptic structure (69–71). This prepatterned mechanisms could also explain the upregulation of the uncoupled nAChRs and SK2 channels in IHCs prior to the efferent innervation during cochlear development and in the adult cochlea following MET current disruption.

### Gene therapy can rescue IHC function and hearing loss in mature mice.

Recent studies have shown that the IHCs of the aging cochlea undergo several progressive changes. In addition to the reemergence of axosomatic efferent synapses (24–26) and a reduced MET current (25), aged IHCs downregulate the Ca^2+^-activated BK channel carrying *I*_K,f_ (40) and upregulate the Ca^2+^-activated SK2 channel *I*_SK2_ (23) in their basolateral membrane (25). These and other morphological and physiological changes cause aged IHCs to closely resemble those present during prehearing stages of development. The mouse line used in this study (*Myo7a^fl/fl^ Myo15-cre^+/–^*) recapitulates most of the changes observed in aged IHCs, thus representing an ideal model to understand the level of plasticity of the mature cochlea and whether it is amenable to therapeutic approaches.

Over the last decade, several advances in the experimental approaches used to correct genetic abnormalities have facilitated the successful delivery of preclinical trials for hearing loss in mice (72, 73). This includes genes involved in IHC exocytosis (*Vglut3*; ref. 74) (*Otof*; refs. 75, 76) and mechanoelectrical transduction (*Tmc1*; refs. 77, 78). However, a recent attempt to rescue inner ear defects in mice with defects in mechanoelectrical transduction due to a nonsense mutation in *Myo7a* (Shaker 1: *Myo7a^4626SB/4626SB^*) using gene-based therapy did largely rescue vestibular defects but not hearing loss (79). The absence of hearing function recovery in *Myo7a^4626SB/4626SB^* mice (79) is most largely due to the highly disorganized stereociliary bundles that characterizes these mice from early stages of development (14). We found that AAV9-*Myo7a* injection in posthearing *Myo7a^fl/fl^ Myo15-cre^+/–^* mice improved the ABR thresholds especially around the 12–16 kHz cochlear region. This is a considerable improvement in hearing function, considering that AAV9 has a very limited transduction efficiency in the OHCs, which reduce hearing thresholds by around 40 dB (80).

In the *Myo7a^fl/fl^ Myo15-cre^+/–^* mice transduced with AAV9-*Myo7a*, IHCs reexpressing MYO7a showed upregulation of the adult-type BK channels and downregulation of the immature-type SK2 channels, indicating that the cells were able to transition back to their characteristic mature phenotype. Since SK2 channels are essential for the establishment of functional efferent synapses (28), our result also indicates that IHCs reestablish their normal mature efferent responses. These findings demonstrate that the adult cochlea is more plastic than originally believed, highlighting gene-based therapy as a realistic approach to prevent or even treat age-related hearing loss.

## Methods

### Sex and biological variable.

We used animals of either sex, which were randomly assigned to the different experimental groups. No statistical methods were used to define sample size, which was defined based on previously published work. Animals were taken from several breeding pairs over several months. Most of the electrophysiological and morphological (but not imaging) experiments were performed in a blinded manner toward animal genotype, and in most cases, experiments were replicated at least 3 times.

### Anesthesia protocols.

For in vivo ABRs, mice were anesthetized using i.p. injection of ketamine (100 mg/Kg body weight) and xylazine (10 mg/Kg, Rompun 2%). At the end of the ABR recordings, mice were either culled by a schedule 1 method (cervical dislocation followed by decapitation) for ex vivo experiments or recovered from anesthesia with injection of atipamezole (1 mg/Kg). For in vivo gene delivery, mice were anesthetized with isoflurane (2.5%) under oxygenation (0.8%). Mice under anesthesia had their body temperature maintained using a heating mat. During the recovery from anesthesia, mice were returned to their cage, placed on a heating mat, and monitored over the following 2–4 hours.

### Animal strains.

For the *Myo7a* and *Otoferlin* (*Otof*) conditional KO mice, the targeted *tm1a* allele (*Myo7a^tm1a(EUCOMM)Wtsi^* allele ID: 4431921; *C57BL/6N-Otof^tm1a(KOMP)Wtsi^* allele ID: 4363757) were generated by the Mouse Genetics Programme at the Wellcome Trust Sanger Institute. The *tm1c* allele was obtained by crossing the *tm1a* mouse to a FLPeR carrying mouse (Rosa26Fki). The *tm1d* alleles, which were used for the experiments, were obtained by crossing the *tm1c* mouse (*Myo7a^fl/fl^* and *Otof^fl/fl^*) with 1 of the following 3 *cre* lines. *Myo15-cre* were donated by Christine Petit (Institut Pasteur, France) and were generated as previously described (27). *Otof*-Cre mice were supplied by Ulrich Mueller (Johns Hopkins University, Baltimore, Maryland, USA) and are now available at MMRRC (https://www.mmrrc.org/catalog/sds.php?mmrrc_id=32781). The tamoxifen inducible *vGlut3-P2A-iCreER*–knock-in mice were donated by Zhiyong Liu (Institute of Neuroscience, Shanghai, China) (43) and are now available at The Jackson Laboratory [B6(Cg)-*Slc17a8^em1(icre/ERT2)Zyliu^*/J, JAX stock no. 034736]. All mouse lines used were on the C57BL/6 background. Both males and females were used for this work.

### AAV production.

The full-length coding sequence of the murine *Myo7a* cDNA (NM_001256081.1) was divided into a 5′ fragment (nucleotides 1–3375) and a 3′ fragment (nucleotides 3376–6648). The 5′ construct contained the 5′ part of the Myo7a cDNA (encoding amino acids 1–1125) and a splice donor (SD) site; the 3′ construct contained the 3′ part of the Myo7a cDNA (encoding amino acids 1126–2215) and a splice acceptor (SA) site. The 5′ construct was generated by inserting a 267 bp fragment containing exons 24 and 25 (synthetized by Genscript) into the p0101_NterMYO7a. To generate the 3′ construct, a synthetic fragment of 1,653 bp was synthetized (Genscript) and cloned into p0101_CterMyo7a, which was digested by EagI/HindIII. Both 5′ and 3′ constructs contain the alkaline phosphatase recombinogenic bridging sequence. The recombinant vectors were packaged into the AAV PHP.eB capsid (ETH Vector Core facility, Zurich). AAV titers were given in viral genomes per mL (vg/mL) as determined by fluorometric assay and was: AAV9-PHP.eB-MYO7A-Nterm (1.5 × 10^13^ vg/mL) and AAV9-PHP.eB-MYO7A-Cterm (6.8 × 10^12^ vg/mL). Final concentrated AAV vector stocks were stored in PBS with MgCl_2_ (1 mM) and KCl (2.5 mM) at –80°C.

### AAV gene delivery in vivo.

The surgical procedure was performed under anesthesia. The right ear was accessed via an incision just below the pinna as previously described (75). When the RWM was identified, it was gently punctured with a borosilicate pipette. This was followed by the injection of 2–3 μL of the AAV into the cochlea. Following the injection, the pipette was retracted from the RWM and the wound was closed with veterinarian glue.

### Tamoxifen injections.

The tamoxifen (T5648, Sigma-Aldrich) was dissolved in corn oil (C8267, Sigma-Aldrich) at a concentration of 20 μg/μl (30°C for 15-–20 minutes) using a sonicator (Brasonic 2510-E, 100W power) and at room temperature (RT). The *Otof^fl/fl^Vglut3-cre^+/–^* mice received 2 i.p. injections of tamoxifen (0.1 mg/gr), the first at P10 and the second 24 hours later. After tamoxifen injections, mice were weighed and monitored daily for up to 5 days for any adverse effects (humane endpoint: weight loss over 20% of the starting body weight).

### ABRs.

Following the onset of anesthesia and the loss of the retraction reflex with a toe pinch, mice were placed in a soundproof chamber (MAC-3, IAC Acoustic). ABRs were recorded from male and female mice placed on a heated mat (37°C). Two subdermal electrodes were placed under the skin behind the pinna of each ear (reference and ground electrode), and 1 electrode was placed half-way between the 2 pinna on the vertex of the cranium (active electrode). Stimuli were presented to the mouse ear by a loudspeaker (MF1-S, Tucker-Davis Technologies) placed 10 cm from the animal’s pinnae, which was calibrated with a low-noise microphone probe system (ER10B+, etymotic). Experiments were performed using customized software driving an RZ6 auditory processor (Tucker-Davis Technologies). Response thresholds were estimated from the resulting ABR waveform, defined as the lower sound level at which any recognizable feature of the waveform was visible. Responses were recorded for pure tones of frequencies and clicks. Stimulus SPLs were 0–95 dB SPL or 0–120 dB SPL, which were presented in steps of 5 dB SPL, and brainstem response signals were averaged over 256 repetitions. Tone bursts were 5 ms in duration with a 1 ms on/off ramp time, presented at a rate of 42.6/sec.

### DPOAEs.

DPOAEs were performed in a soundproof chamber (MAC-3). DPOAEs were recorded at 2f1–f2 in response to primary tones f1 and f2, where f2/f1 = 1.2. The f2 level (L2) was set from 20 to 80 dB (the maximum level set for our system) in 10 dB increments, and the f1 level (L1) was set equal to L2. Frequency pairs of tones between f2 = 6.5 kHz and f2 = 26.3 kHz were delivered into the left ear canal of mice by using a coupler connected to 2 calibrated loudspeakers (MF1-S). The response signal was averaged over 500 repetitions. The emission signals were recorded by a low-noise microphone (ER10B+) connected to the same ear coupler mentioned above. Experiments were performed using BioSigRZ software driving an RZ6 auditory processor. DPOAE thresholds were defined by the minimal sound level where the DPOAEs were above the SD of the noise using a custom-made software.

### Tissue preparation.

IHCs were studied in acutely dissected cochlear from mice of both sexes. Organs of Corti were dissected using the solution (in mM): 135 NaCl, 5.8 KCl, 1.3 CaCl_2_, 0.9 MgCl_2_, 0.7 NaH_2_PO_4_, 5.6 D-glucose, 10 HEPES-NaOH. Sodium pyruvate (2 mM), amino acids, and vitamins were added from concentrates (Thermo Fisher Scientific) (pH: 7.48; ~308 mmol kg^–1^). The dissected apical coil of the cochlea, which correspond to the 9–12 kHz region, was transferred to a microscope chamber and immobilized with a nylon mesh fixed to a stainless-steel ring and viewed using an upright microscope with a custom-made rotating stage (Olympus BX51). A peristaltic pump (Cole-Palmer) was used to continually perfuse the microscope chamber, and the IHCs were visualized with Nomarski Differential Interference Contrast (DIC) optics (60× water immersion objective, Olympus) and ×15 eyepieces.

### Single-cell electrophysiology.

Patch-clamp recordings of basolateral K^+^ currents from the IHCs were performed as previously described (15, 25) either at RT (20°C–25°C) or body temperature (34°C–37°C). Patch pipettes with resistances of 2–3 MΩ were pulled from soda glass capillaries (Harvard Apparatus Ltd.), and the shanks of the electrodes were coated with surf wax (Mr. Zog’s Cool Water Sex Wax) to reduce the electrode capacitative transient. The patch pipette intracellular solution contained (in mM): 131 KCl, 3 MgCl_2_, 1 EGTA-KOH, 5 Na_2_ATP, 5 HEPES-KOH, and 10 Na-phosphocreatine (pH 7.28; 294 mmol kg^–1^). Membrane current and voltage responses were obtained using an Optopatch amplifier (Cairn Research Ltd.). Data acquisition was controlled by pClamp software using a Digidata 1440A (Molecular Devices). Recordings were low-pass filtered at 2.5 kHz (8-pole Bessel), sampled at 5 kHz, and stored on computer for off-line analysis (Origin). Membrane potentials were corrected for the residual series resistance (*R_s_*) after compensation (usually 80%) and liquid junction potential (LJP) of –4 mV.

IPSCs in IHCs were obtained by superfusing a 40 mM K^+^ solution to depolarize the efferent fibers. Additional extracellular solutions containing 100 μM ACh (A6625, Sigma-Aldrich) alone or in the presence of 1 μM of the nAChR blocker strychnine (Sigma-Aldrich) were applied by a gravity-fed multichannel pipette positioned close to the IHCs.

Real-time changes in *C*_m_ (Δ*C*_m_) were performed at body temperature as previously described (47, 48). Briefly, a 4 kHz sine wave of 13 mV RMS was applied to IHCs from −81 mV, which was interrupted for the duration of the voltage step. The capacitance signal from the Optopatch was filtered at 250 Hz and sampled at 5 kHz. Δ*C*_m_ was measured by averaging the *C*_m_ trace over a 200 ms period following the voltage step and subtracting the prepulse baseline. The intracellular solution used for these experiments contained (in mM): 106 Cs-glutamate, 20 CsCl, 3 MgCl_2_, 1 EGTA-CsOH, 5 Na_2_ATP, 0.3 Na_2_GTP, 5 HEPES-CsOH, and 10 Na_2_-phosphocreatine (pH 7.3; 294 mOsm kg^–1^). The extracellular solution for *ΔC*_m_ recordings contained 30 mM TEA, 15 mM 4-AP (Fluka), and 80 μM linopirdine (Tocris) to block the BK current (*I*_K,f_) (40), the delayed-rectifier K^+^ current (*I*_K_), and negatively activating K^+^ current (*I*_K,n_), respectively. Membrane potentials were corrected for the voltage drop across the residual *R*_s_ and a LJP of –11 mV.

For MET current recordings, the IHC hair bundles were displaced using a fluid jet from a borosilicate glass pipette driven by 25 mm diameter piezoelectric disc. Mechanical stimuli were applied as 50 Hz sinusoids (filtered at 1 kHz, 8-pole Bessel).

### Immunofluorescence microscopy.

For prehearing mice, the inner ear was immersed in 4% paraformaldehyde in phosphate-buffered saline (PBS, pH 7.4) for 20 minutes at RT. For adult mice, the inner ear was initially gently perfused with the above solution for 1–2 minutes through the round window following the gentle opening of the bone on the apical coil of the cochlea. Following this initial brief fixation, the adult inner ear was fixed for a further 20 minutes at RT. After the fixation, the cochleae were rinsed 3 times in PBS for 10 minutes at RT, and the organs of Corti were dissected out. The organ of Corti was then incubated for 1 hour at RT in PBS supplemented with 5% normal horse serum (H0146, MilliporeSigma) and 0.5% Triton X-100 (T9284, MilliporeSigma). The samples were then incubated overnight at 37°C with the primary antibodies in PBS supplemented with 1% normal horse serum. Primary antibodies were as follows: mouse IgG1 anti-ATP1A3 (Na^+^/K^+^ ATPase 3, 1:500, Thermo Fisher Scientific, MA3915), mouse anti–myosin 7a (1:100, Developmental Studies Hybridoma Bank, 138-1C), rabbit anti–myosin 7a (1:500, Proteus Biosciences, 25-6790), rabbit anti-SK2 (1:200, MilliporeSigma, P0483), goat anti–choline acetyltransferase (ChAT, 1:500, MilliporeSigma, AB144P), mouse IgG1 anti-CtBP2 (1:500, BD Biosciences, 612044), mouse IgG2a anti-GluR2 (1:200, MilliporeSigma, MAB397), mouse anti-otoferlin (1:2000, Abcam, ab53233), and mouse anti-BK (1:500, Antibodies Incorporated, 75-408). The following day, the samples were rinsed 3 times with PBS for 10 minutes at RT before being labeled with species specific Alexa Fluor secondary antibodies (1:1,000) for 1 hour at 37°C. Samples were rinsed a final 3 times (10 minutes, RT) before being mounted on glass slides in VECTASHIELD (H-1000, Vector Laboratories).

For sequential immunolabeling experiments, samples were fixed as above and incubated for 1 hour at RT in PBS supplemented with 5% normal horse serum and 0.5% Triton X-100. Samples were incubated with a primary antibody (rabbit anti-SK2; 1:200) overnight at 37°C. The following day, the samples were rinsed 3 times with PBS for 10 minutes at RT and labeled with species specific Alexa Fluor secondary antibodies for 1 hour at 37°C. Samples were then washed 3 times with PBS (10 minutes, RT) before being incubated with PBS supplemented with 5% normal horse serum and 0.5% Triton X-100. The primary antibody (rabbit anti–myosin 7a; 1:500) was then incubated in PBS supplemented with 5% normal horse serum for 2 hours at 37°C, followed by 3 washes in PBS (10 minutes, RT). Samples were then incubated with an appropriate species-specific Alexa Fluor secondary antibody (1:1,000) in a PBS supplemented with 5% normal horse serum for 1 hour at 37°C. Lastly, the samples were rinsed a final 3 times in PBS (10 minutes, RT) and mounted on glass slides in VECTASHIELD.

The *Z* stack images were captured on either a Zeiss LSM 880 with AiryScan for superresolution confocal microscopy (Plan-Apochromat 63×/ Oil DIC M27 objective; numerical aperture, 1.4) or on a Nikon CSU-W1 Spinning disk confocal microscope with either a 25 μm or 50 μm pinhole size (either: Plan-Apochromat 20×, N.A. 0.75; 40× oil, N.A. 1.3; 100× oil, N.A. 1.45). Image stacks were processed with Fiji ImageJ (NIH) analysis software.

### Statistics.

Statistical comparisons of means were made by Student’s 2-tailed *t* test, Mann-Whitney *U* test, or, for multiple comparisons, ANOVA (1-way or 2- way ANOVA followed by a suitable post hoc test). *P* < 0.05 was the selected criterion for statistical significance. Averages are shown within the text and figures as means ± SD.

### Study approval.

All animal work was performed at the University of Sheffield, licenced by the Home Office under the Animals (Scientific Procedures) Act 1986 (PCC8E5E93 and PP1481074) and approved by the University of Sheffield Ethical Review Committee (180626_Mar).

### Data availability.

All data are available from the Supporting Data Values file or from the corresponding author upon request.

## Author contributions

APO, AEA, AZ, SAH, AJC, FK, MSR, JYJ, MJL, SLJ, SS, and WM helped with the collection and analysis of the data. WM conceived and coordinated the study. APO, AEA, AZ, SAH, AJC, FK, MSR, JYJ, MJL, SLJ, SS, and WM approved the final version of the manuscript. APO, AEA, AZ, SAH, AJC, FK, MSR, JYJ, MJL, SLJ, SS, and WM agree to be accountable for all aspects of the work in ensuring that questions related to the accuracy or integrity of any part of the work are appropriately investigated and resolved. All persons designated as authors qualify for authorship, and all those who qualify for authorship are listed.

## Supplementary Material

Supplemental data

Supporting data values

## Version 1 12/06/2024

Electronic publication
