# Supplementary material for: In vivo AAV9-Myo7a gene rescue restores hearing and cholinergic efferent innervation in inner hair cells
Source: JCI Insight. 2024 Dec 6;9(23):e182138. doi: 10.1172/jci.insight.182138 (PMC11623941; doi:10.1172/jci.insight.182138)
Supplement: Supplemental data [file jciinsight-9-182138-s219.pdf]

In vivo AAV9-_Myo7a_ gene rescue restores hearing and cholinergic efferent innervation in inner hair cells.

Andrew O'Connor, Ana E. Amariutei, Alice Zanella, Sarah Hool, Adam J. Carlton, Fanbo Kong, Mauricio Saenz Roldan, Jing-Yi Jeng, Marie José Lecomte, Stuart L. Johnson, Saaid Safieddine, Walter Marcotti

This document includes 10 Supplementary Figures.

Supplementary Figure 1

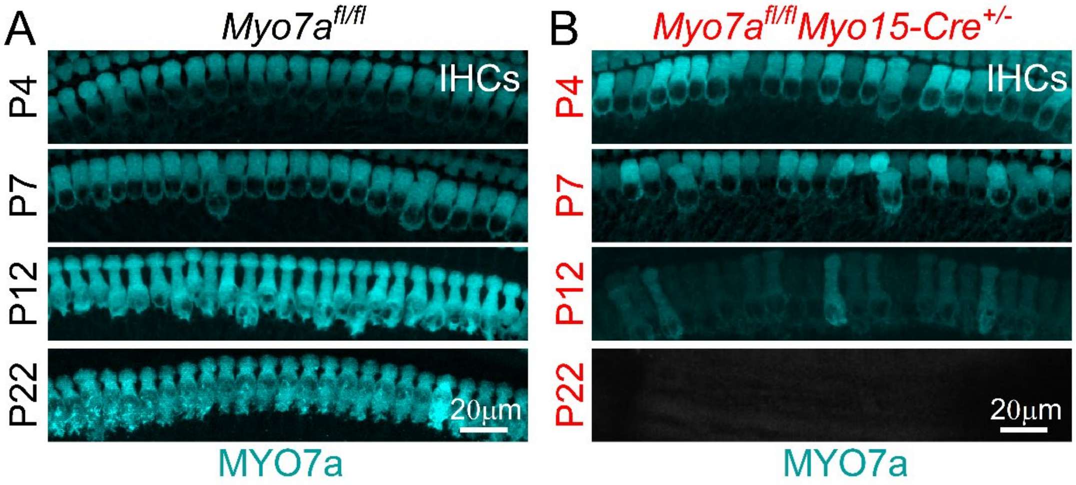

Supplemental Figure 1: Expression of *Myo7a* in pre- and post-hearing *Myo7a*$^{fl/fl}$*Myo15-cre*$^{+/-}$ mice

(**A,B**) Cochlear whole mount preparations from *Myo7a*$^{fl/fl}$ (**A**) and *Myo7a*$^{fl/fl}$*Myo15-cre*$^{+/-}$ (**B**) mice at pre-hearing ages (P4, P7), at around the onset of hearing (P12) and in the mature cochlea (P22) immunostained for MYO7a. At P12, MYO7A expression was already largely reduced and was no longer present at P22. At least 3 mice per genotype were used.

Supplemental Figure 2

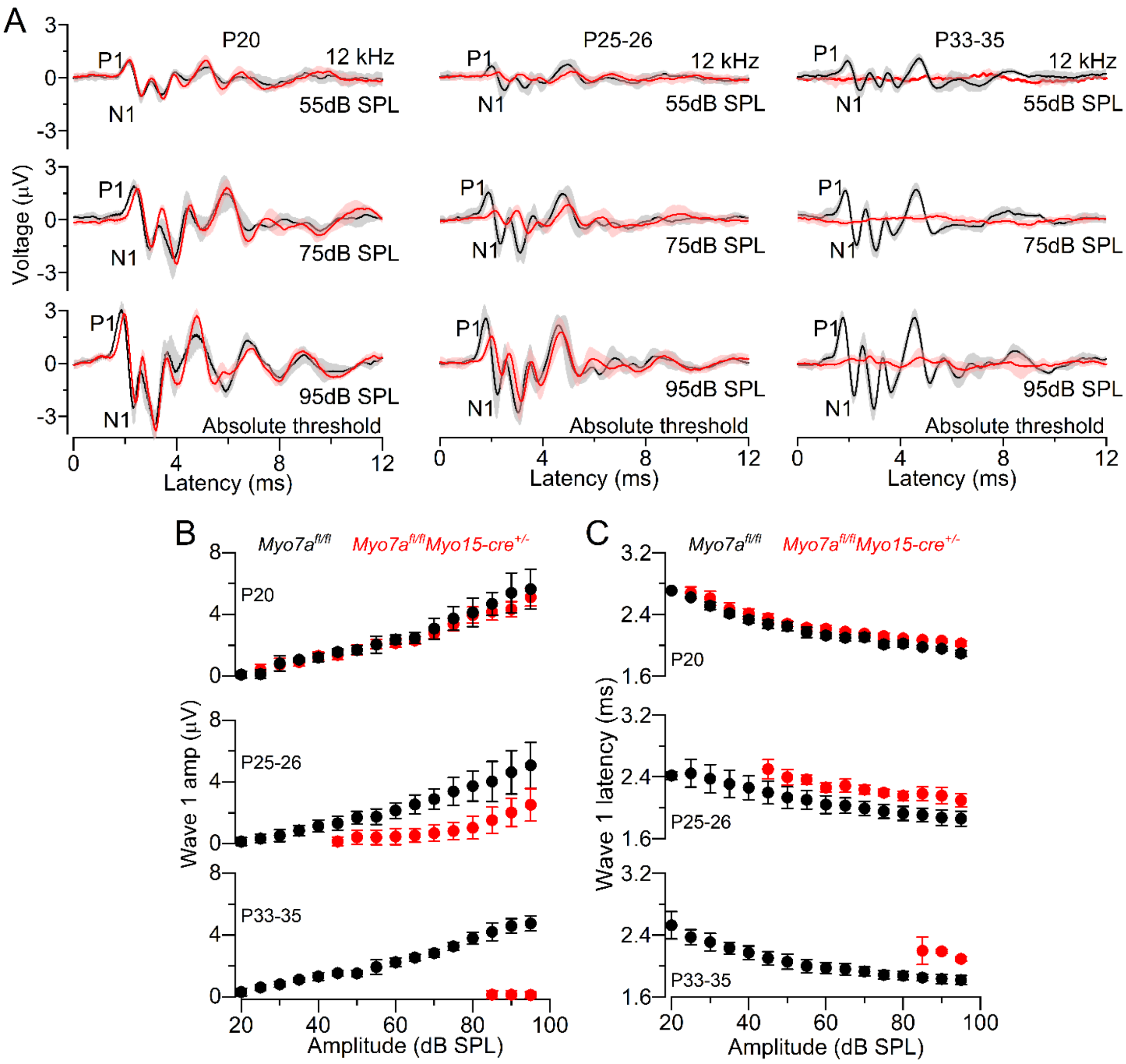

Supplemental Figure 2: ABR waveforms evoked in *Myo7a$^{fl/fl}$Myo15-Cre$^{+/-}$* mice
(**A**) Average ABR waveform responses at 12 kHz and different sound intensities (55, 75 and 95 dB SPL) for the different age ranges at which we could reliable measure them in both control and littermate *Myo7a$^{fl/fl}$ Myo15-cre$^{+/-}$* mice. Continuous lines represent average values and shaded areas the SD. P1 and N1 indicate the positive and negative peaks of wave I. (**B**) Average amplitude of wave I (from P1 to N1) as a function of age for control and *Myo7a$^{fl/fl}$ Myo15-cre$^{+/-}$* mice at different sound intensities. For P31-35 age group, *Myo7a$^{fl/fl}$ Myo15-cre$^{+/-}$* mice only responded at the upper threshold limit of our system. Statistical comparison for the overlapping range between the two genotypes: P = 0.0010 for P20; P < 0.0001 for both P25-26 and P31-P35 ranges (two-way ANOVA). (**C**) Average latency of wave I (time between stimulus onset and P1) as a function of age for control and *Myo7a$^{fl/fl}$ Myo15-cre$^{+/-}$* mice at different sound intensities. Statistical comparisons: P< 0.0001 for all age ranges (two-way ANOVA). Values in **B** and **C** are mean ± SD. Number of mice are as listed in **Figure 1**. Note that when wave I amplitude is not visible (i.e., amplitude 0µV), the latency was not measured.

Supplemental Figure 3

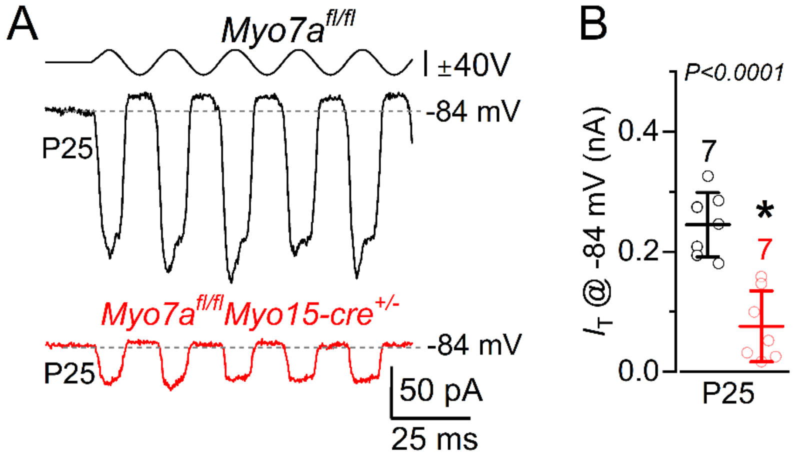

Supplemental Figure 3: Mechanoelectrical transduction in OHCs from adult *Myo7a*$^{fl/fl}$ mice. (**A**) Saturating MET currents recorded from apical-coil OHCs of P25 control (top: *Myo7a*$^{fl/fl}$) and littermates *Myo7a* knockout mice (bottom: *Myo7a*$^{fl/fl}$*Myo15-cre*$^{+/-}$). MET currents were elicited using 50 Hz sinusoidal force stimuli to the hair bundles at the membrane potential of –84 mV. Driver voltage (DV) stimuli to the fluid jet are shown above the traces, with positive deflections of the DV being excitatory. (**B***)* Average peak to peak MET current-voltage curves from apical OHCs of control and littermate *Myo7a*$^{fl/fl}$*Myo15-cre*$^{+/-}$ P25 mice. Data are shown as mean ± SD. Number of mice used is shown above the data points. Statistical value shown is from *t*-test.

Supplemental Figure 4

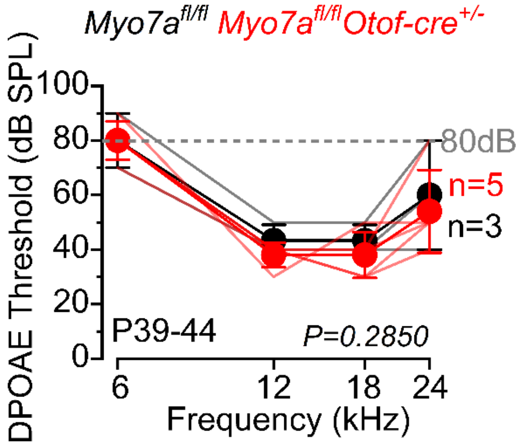

Supplemental Figure 4. *Myo7a$^{fl/fl}$Otof-cre$^{+/-}$* does not affect OHC function.
Average distortion product otoacoustic emission (DPOAE) thresholds for frequency-specific pure tone burst stimuli at 6, 12, 18 and 24 kHz recorded from control *Myo7a$^{fl/fl}$* (black) and littermate *Myo7a$^{fl/fl}$Otof-cre$^{+/-}$* (red) mice at P39-44. Single DPOAE recordings from individual animals are also shown (*Myo7a$^{fl/fl}$*: grey lines; *Myo7a$^{fl/fl}$Otof-cre$^{+/-}$*: pink lines). Stated significance value is from two-way ANOVA. Data are shown as mean ± SD.

Supplemental Figure 5

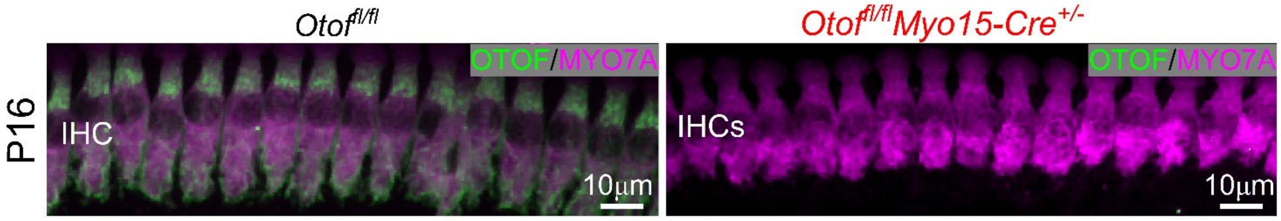

Supplemental Figure 5. Otoferlin in the IHCs from *Otof*$^{fl/fl}$*Myo15-cre*$^{+/-}$ is downregulated by P16.

Maximum intensity projections of confocal z-stack images taken from the 9-12 kHz apical region of the cochlea in *Otof*$^{fl/fl}$ (**C**) and *Otof*$^{fl/fl}$*Myo15-cre*$^{+/-}$ mice at P16. Cochleae were labelled with antibodies against Otoferlin (green) and the IHC marker MYO7A (magenta). Note that otoferlin is missing in the IHCs from *Otof*$^{fl/fl}$*Myo15-cre*$^{+/-}$ mice. Three mice per genotype were used for immunostaining experiments. Scale bars: 10μm.

Supplemental Figure 6

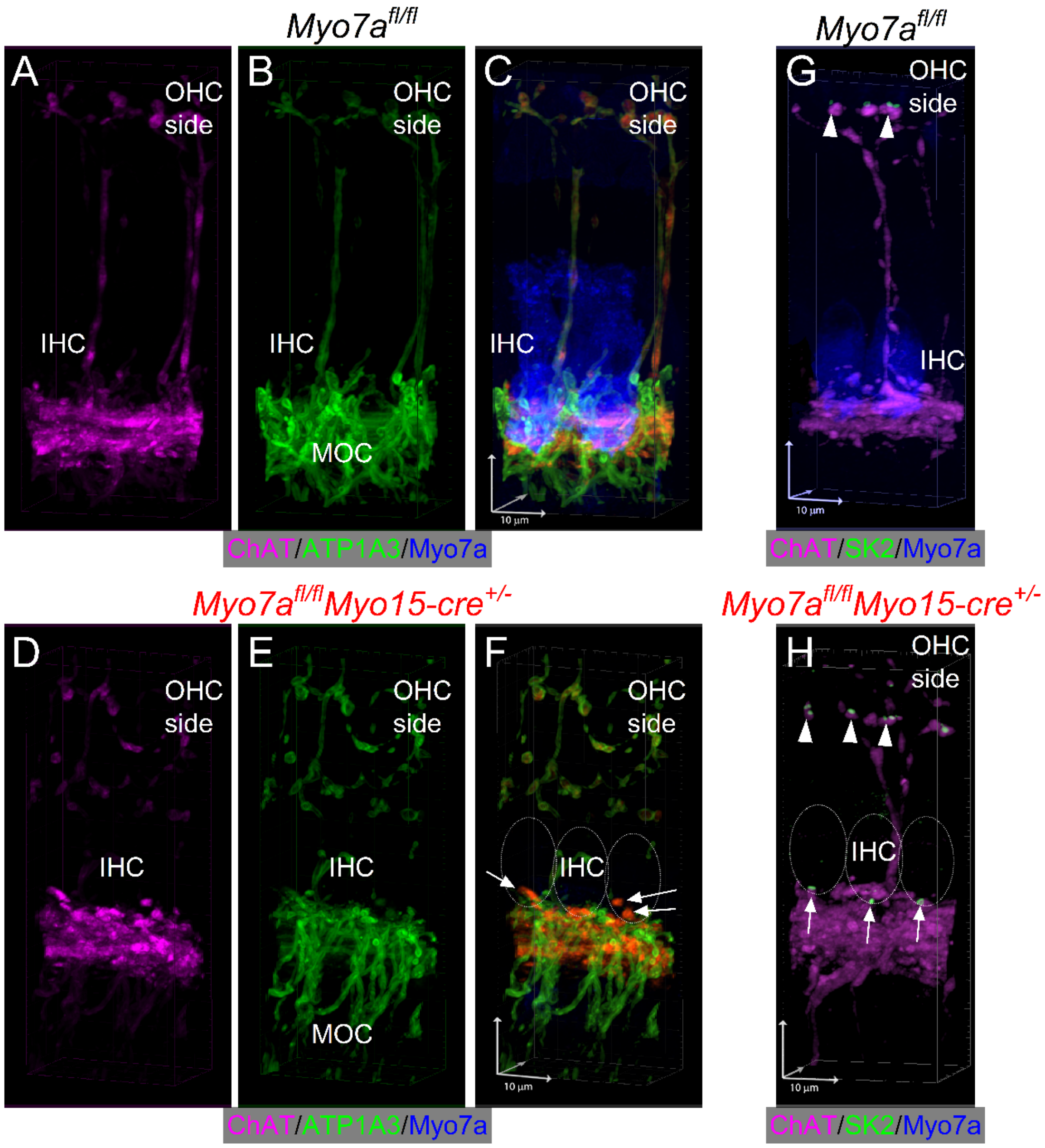

Supplemental Figure 6. Efferent LOC terminals make axo-somatic contacts with IHCs from *Myo7a$^{fl/fl}$Myo15-cre$^{+/-}$* mice.

A-F, Confocal z-stacks taken from the apical cochlea of 1 month old control *Myo7a$^{fl/fl}$* (**A-C**) and *Myo7a$^{fl/fl}$Myo15-cre$^{+/-}$* (**D-F**) mice, labelled with antibodies against ChAT (***A***: magenta), ATP1A3 (***B***: green) and Myo7a (blue, hair cell marker). Panel **C** and **F** shows the superimposed ChAT (magenta), ATP1A3 (green) and Myo7a (blue) labelling. Because *Myo7a$^{fl/fl}$Myo15-cre$^{+/-}$* do not express MYO7A, the borders of the 3 IHCs have been indicated by a dash line. Images highlight that the efferent terminal on OHCs are both ChAT and ATP1A3 positive in both genotypes. However, ChAT positive puncta but negative for the MOC marker ATP1A3, were present on the IHCs of *Myo7a$^{fl/fl}$Myo15-cre$^{+/-}$* mice (**F**, arrows). **G,H**, Confocal z-stacks from control *Myo7a$^{fl/fl}$* (**G**) and *Myo7a$^{fl/fl}$Myo15-cre$^{+/-}$* (**H**) mice, labelled with antibodies against ChAT (***A***: magenta), SK2 (***B***: green) and Myo7a (blue, hair cell marker). Note that in *Myo7a$^{fl/fl}$Myo15-cre$^{+/-}$* mice (**H**) IHCs have SK2 puncta are next to ChAT labelling, indicating the presence of post-synaptic efferent synapses. At least 3 mice were used for each genotype and condition.

Supplemental Figure 7

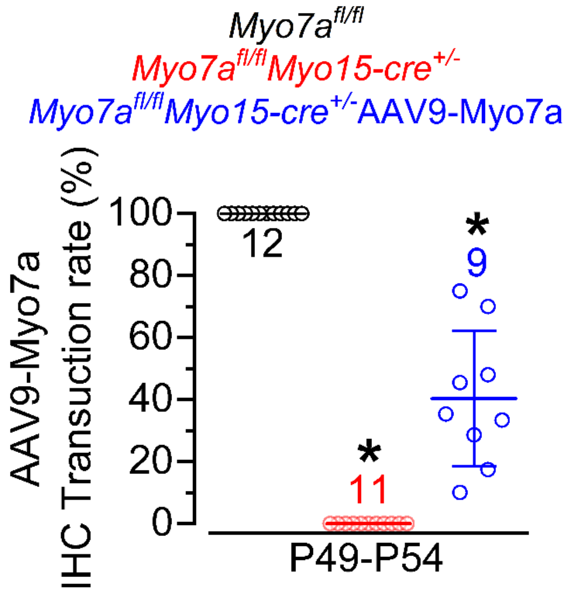

Supplemental Figure 7. Transduction efficiency of AAV9-*Myo7*a in targeting the IHCs of the adult mouse cochlea.

Viral-transduction rates in apical IHCs determined from the number of MYO7A-positive cells identified via immunostaining in each cochlear preparation over a 150 µm range from control *Myo7a*$^{fl/fl}$ (black), *Myo7a*$^{fl/fl}$*Myo15-cre*$^{+/-}$ (magenta) and *Myo7a*$^{fl/fl}$*Myo15-cre*$^{+/-}$ injected with AAV9-MYO7A (blue) at P49-54. Note that on average AAV9-*Myo7a* transduces about 40% of the IHCs. All comparisons were found to be significantly different. Data are shown as mean ± SD. The values above or below the average data represent the number of mice. $P < 0.0001$, Tukey's post-test, one-way ANOVA. Number of mice tested is shown above the data.

Supplemental Figure 8

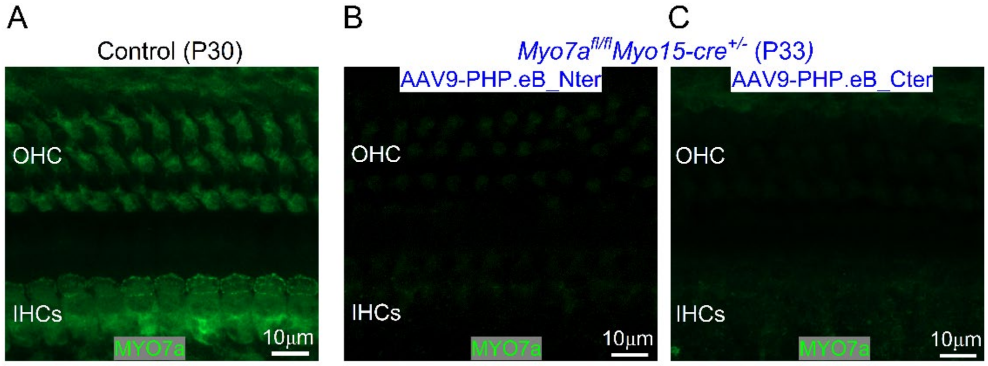

Supplemental Figure 8. Single AAV9-Myo7a Nterm and CTerm do not induce the expression of MYO7A in hair cells.
(**A-C**) Maximum intensity projections of confocal z-stack images taken from the apical region of the cochlea in non-injected control (**A**) and *Myo7a*$^{fl/fl}$*Myo15-cre*$^{+/-}$ injected with either the AAV9-PHP.eB-MYO7A-Nter (**B**) or AAV9-PHP.eB-MYO7A-Cter (**C**). Note that MYO7A was not present in the hair cells of *Myo7a*$^{fl/fl}$*Myo15-cre*$^{+/-}$ when the N-terminal and the C-terminal of the AAV9-PHP.eB-Myo7A were injected separately.

Supplemental Figure 9

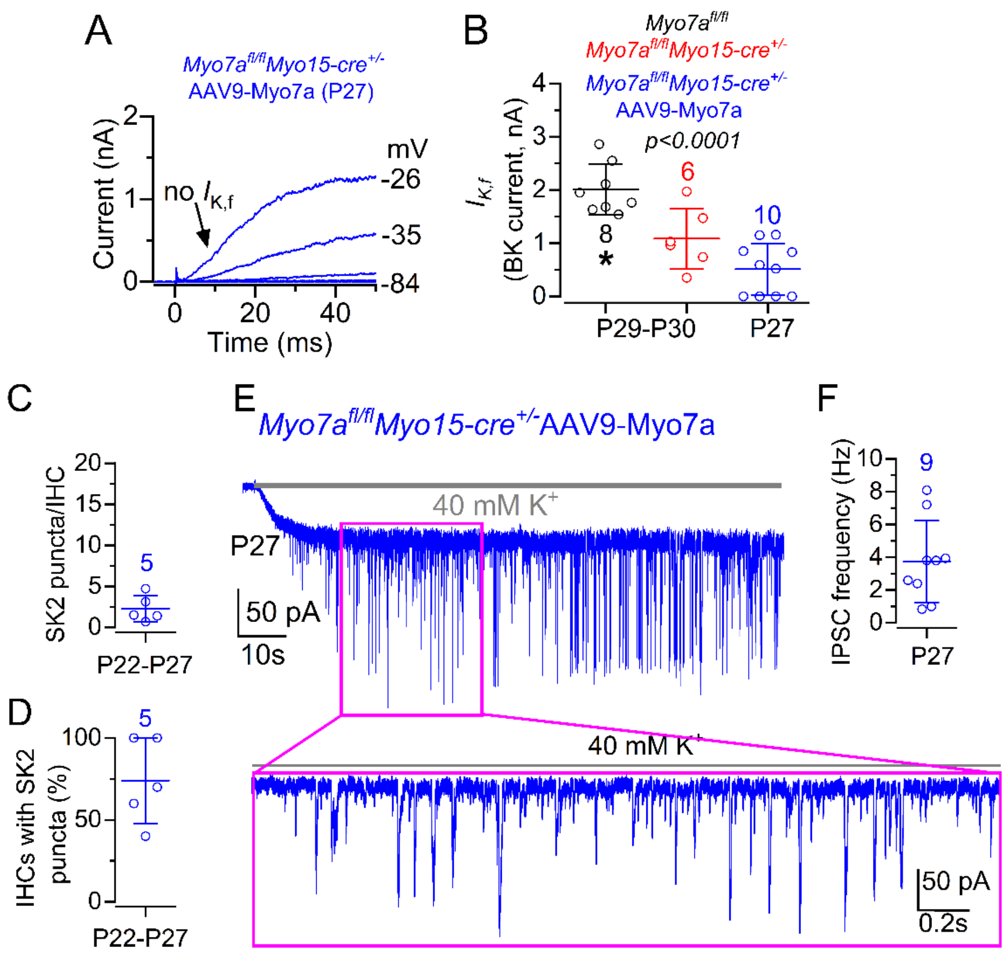

Supplemental Figure 9. Size of $I_{K,f}$, SK2 expression and efferent synaptic activity in young adult IHCs from *Myo7a$^{fl/fl}$Myo15-cre$^{+/-}$* injected with AAV9-MYO7A.

(**A**) Current responses from an IHCs in a P27 *Myo7a$^{fl/fl}$Myo15-cre$^{+/-}$* mouse transduced with AAV9-*Myo7a* at P13. Current recordings were elicited by using depolarizing voltage steps (10 mV increments) from the holding potential of –84 mV to the various test potentials shown by some of the traces. Although adult IHCs normally express the rapid activating outward K^+ current $I_{K,f}$ carried by BK channels, it was still absent in IHCs from young adult *Myo7a$^{fl/fl}$Myo15-cre$^{+/-}$* mice transduced with AAV9-*Myo7a*. (**B**) Average size of the isolated $I_{K,f}$, which was measured at 1 ms from the stimulus onset and at -25 mV. Values in blue are those recorded from IHCs of *Myo7a$^{fl/fl}$Myo15-cre$^{+/-}$* mice transduced with AAV9-*Myo7a*, which also include the recording shown in panel (**A**). The values in red (*Myo7a$^{fl/fl}$Myo15-cre$^{+/-}$*) and black (control *Myo7a$^{fl/fl}$*) are from **Reference [15]**. Number of mice used is shown above the data points. Statistical significance value is from one-way ANOVA. (**C,D**) Number of SK2 puncta per IHC (**C**) and percentage of IHCs that showed SK2 puncta (**D**) in 150 µm of the apical cochlea region of P22-P27 *Myo7a$^{fl/fl}$Myo15-cre$^{+/-}$* mice injected with AAV9-Myo7a at P13. The number of mice used is shown above the data. (**E**) Inward membrane currents recorded from an IHC of a P27 *Myo7a$^{fl/fl}$Myo15-cre$^{+/-}$* mouse transduced with AAV9-Myo7a at P13. Currents were elicited by using 40 mM extracellular KCl as described in Figure 3. (**F**) Frequency of IPSCs recorded from nine P27 IHCs from three *Myo7a$^{fl/fl}$Myo15-cre$^{+/-}$* mice transduced with AAV9-*Myo7a*. Data in this figure are shown as mean ± SD.

Supplemental Figure 10

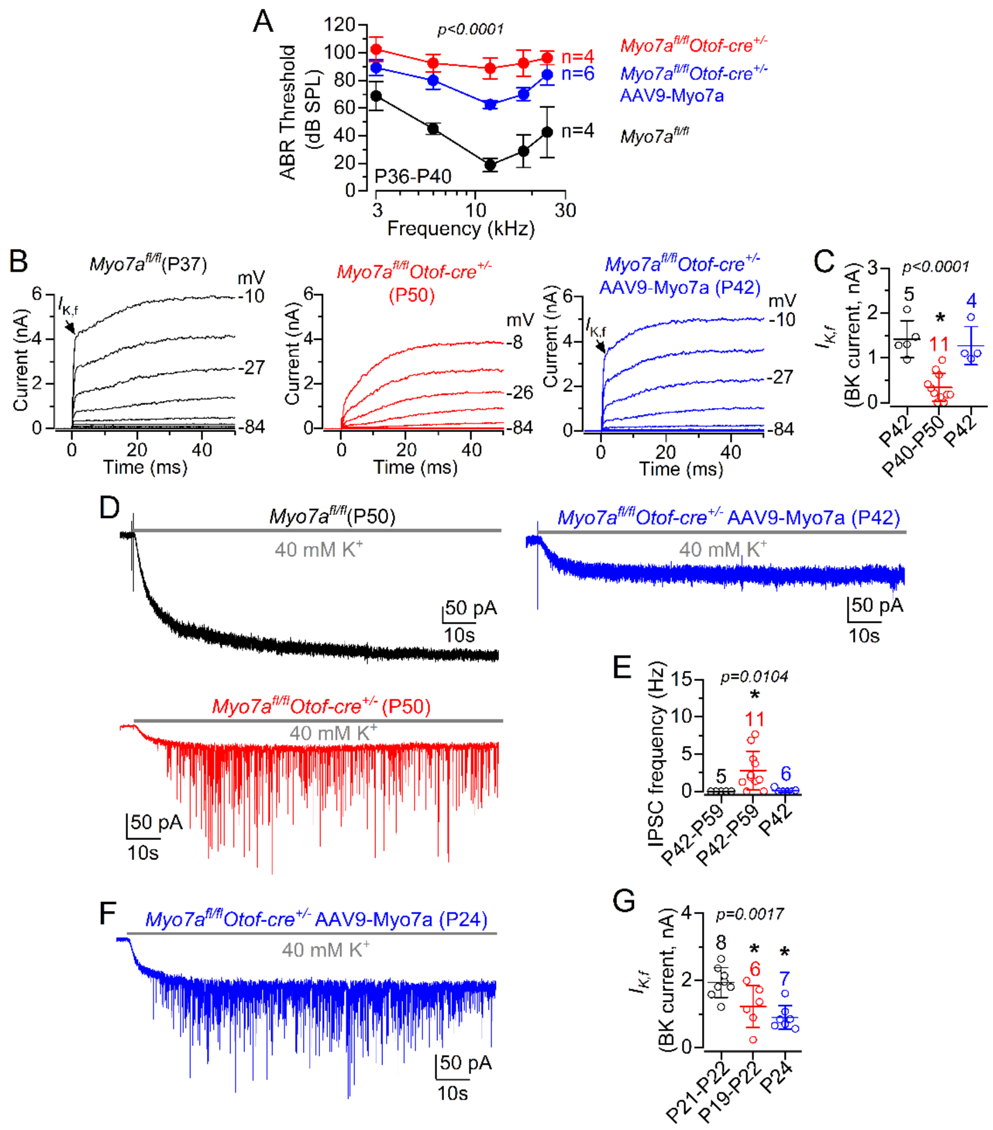

Supplemental Figure 10. Partial functional recovery of adult *Myo7a*$^{fl/fl}$*Otof-cre*$^{+/-}$ injected with AAV9-MYO7A.

(**A**) Average ABR thresholds for frequency-specific pure tone burst stimuli recorded from control *Myo7a*$^{fl/fl}$ (black), *Myo7a*$^{fl/fl}$*Otof-cre*$^{+/-}$ (red) and *Myo7a*$^{fl/fl}$*Otof-cre*$^{+/-}$ injected with AAV9-*Myo7a* (blue) mice. The number of mice used for each genotype is shown near the data. The dashed lines indicate the upper threshold limit used for this experiment (120 dB SPL). Statistical comparison is from two-way ANOVA (post-test: $P < 0.0001$ for all comparisons). (**B**) Example of outward K^+ current responses from IHCs of the control (*Myo7a*$^{fl/fl}$, black), *Myo7a*$^{fl/fl}$*Otof-cre*$^{+/-}$ (red) and *Myo7a*$^{fl/fl}$*Otof-cre*$^{+/-}$ mice injected with AAV9-*Myo7a* (Blue). Currents were elicited using depolarizing voltage steps (10 mV increments) from the holding potential of –84 mV to the various test potentials shown by some of the traces. Note that the large $I_{K,f}$ (arrow), which is carried by BK channels and is characteristic of mature IHCs, is only present in control (black) and *Myo7a*$^{fl/fl}$*Otof-cre*$^{+/-}$ mice injected with AAV9-*Myo7a* (blue). (**C**) Size of the isolated current $I_{K,f}$ measured at -25 mV and at 1 ms from the voltage step onset in the three experimental conditions shown in panel (**B**). The number of IHCs tested is shown above the data; number of mice from left to right: 2, 4 and 2. $I_{K,f}$ was significantly reduced in *Myo7a*$^{fl/fl}$*Otof-cre*$^{+/-}$ compared to both controls and AAV9-*Myo7a* injected mice ($P < 0.0001$ and $P = 0.0010$, respectively, Tukey's post-test from one-way ANOVA). No significant difference was found between *Myo7a*$^{fl/fl}$ and *Myo7a*$^{fl/fl}$*Otof-cre*$^{+/-}$ mice injected with AAV9-*Myo7a* ($P = 0.8203$). (**D**) Inward membrane currents recorded from IHCs of *Myo7a*$^{fl/fl}$ (black), *Myo7a*$^{fl/fl}$*Otof-cre*$^{+/-}$ (red) and *Myo7a*$^{fl/fl}$*Otof-cre*$^{+/-}$ mice injected with AAV9-*Myo7a* (blue).

Recording protocol is as described in **Figure 3**. (**E**) Average frequency of the IPSCs recorded from IHCs of 3 *Myo7a*$^{fl/fl}$, 5 *Myo7a*$^{fl/fl}$*Otof-cre*$^{+/-}$ and 2 *Myo7a*$^{fl/fl}$*Otof-cre*$^{+/-}$ mice injected with AAV9-*Myo7a*. The frequency of IPSCs was significantly reduced in both *Myo7a*$^{fl/fl}$ and *Myo7a*$^{fl/fl}$*Otof-cre*$^{+/-}$ mice injected with AAV9-*Myo7a* compared to *Myo7a*$^{fl/fl}$*Otof-cre*$^{+/-}$ (P = 0.0311 and P = 0.0285, respectively). No significant difference was found between *Myo7a*$^{fl/fl}$ and *Myo7a*$^{fl/fl}$*Otof-cre*$^{+/-}$ mice injected with AAV9-*Myo7a* (P = 0.9932). (**F**) Inward membrane currents recorded from an IHC of a P24 *Myo7a*$^{fl/fl}$*Otof-cre*$^{+/-}$ mouse transduced with AAV9-Myo7a at P13. Currents were elicited by using 40 mM extracellular KCl as described in **Figure 3**. IPSCs were recorded in all 6 IHCs tested, with a frequency of 4.8 ± 3.6 Hz. (**G**) Size of the isolated BK current ($I_{K,f}$) recorded from P19-P24 IHCs of the three different experimental mouse lines. $I_{K,f}$ was significantly reduced in both *Myo7a*$^{fl/fl}$*Otof-cre*$^{+/-}$ and *Myo7a*$^{fl/fl}$*Otof-cre*$^{+/-}$ mice injected with AAV9-*Myo7a* compared to controls (P = 0.0323 and P = 0.0015, respectively). The findings in panels (**F,G**) show that the injection of AAV9-Myo7a in *Myo7a*$^{fl/fl}$*Otof-cre*$^{+/-}$ at P13 did not prevent the normal down-regulation of BK channels and the return of IPSC responses in the absence of MYO7A. Statistical values shown in panels (**C,E,G**) are from one-way ANOVA and average values are shown as mean ± SD.
